# YfiB: An Outer Membrane Protein Involved in the Virulence of *Shigella flexneri*

**DOI:** 10.3390/microorganisms10030653

**Published:** 2022-03-18

**Authors:** Tanuka Sen, Naresh K. Verma

**Affiliations:** Division of Biomedical Science and Biochemistry, Research School of Biology, The Australian National University, Canberra, ACT 2601, Australia; tanuka.sen@anu.edu.au

**Keywords:** *Shigella flexneri*, biofilm formation, YfiBNR signalling, virulence, bacterial invasion, plaque formation, site-directed mutagenesis

## Abstract

The intracellular pathogen *Shigella flexneri*, which is the causative agent of bacillary dysentery, significantly influences the worldwide implication of diarrheal infections, consequentially causing about 1.1 million deaths each year. Due to a nonavailability of an authorized vaccine and the upsurge of multidrug resistance amongst *Shigella* strains, there has been a huge demand for further genetic analyses which could help in the advancement of new/improved drugs, and finding vaccine candidates against the pathogen. The present study aims to illustrate the role of the *yfiB* gene in *Shigella* virulence, part of the periplasmic YfiBNR tripartite signalling system. This system is involved in the regulation of cyclic-di-GMP levels inside the bacterial cells, a vital messenger molecule impacting varied cellular processes such as biofilm formation, cytotoxicity, motility, synthesis of exopolysaccharide, and other virulence mechanisms such as adhesion and invasion of the bacteria. Through a combination of genetic, biochemical, and virulence assays, we show how knocking out the *yfiB* gene can disrupt the entire YfiBNR system and affect the native c-di-GMP levels. We found that this subsequently causes a negative effect on the biofilm formation, bacterial invasion, host–surface attachment, and the overall virulence of *Shigella*. This study also carried out a structural and functional assessment of the YfiB protein and determined critical amino acid residues, essential for proper functioning of this signalling system. The present work improves our understanding of the in vivo persistence and survival of *Shigella*, brings light to the c-di-GMP led regulation of *Shigella* virulence, and provides a prospective new target to design anti-infection drugs and vaccines against *S. flexneri* and other bacterial pathogens.

## 1. Introduction

*Shigella* is responsible for instigating an invasive enteric infection in humans, known as shigellosis, and it does this by aggressively attacking the colonic epithelium [1]. It is a Gram-negative bacterium and a well-studied intracellular pathogen, belonging to the *Enterobacteriaceae* family, closely resembling *E. coli* [1,2]. Globally, shigellosis leads to an enormous amount of morbidity and mortality annually, especially in developing and underdeveloped countries, significantly contributing to the worldwide burden of diarrheal infections [3,4,5].

Out of the four known *Shigella* species, *S. flexneri*, accounts for about 10% of all the diarrhoetic incidents amongst children below 5 years of age in areas of low socioeconomic conditions [6]. In the absence of a globally effective licensed vaccine against *Shigella*, the current treatment method majorly depends on antimicrobial therapy. The treatment with antibiotics is known to be compromised due to many factors such as poor sanitation and hygiene, treatment costs, overuse and/or misuse of antibiotics [7,8,9]. As a result, there is an ever-growing challenge of antimicrobial resistance in *Shigella*, which then leads to the likelihood of a poor outcome in shigellosis cases [7,8,9].

The intracellular lifestyle of *Shigella*, including its invasion process and the immune response it generates in the host, has been comprehensively studied using various in vivo and in vitro assays, which has expanded our understanding of the pathogen [10]. The virulence genes present on the 30 kb entry region of the large virulence plasmid and the chromosomal genes part of the various pathogenicity islands (PAI) play a crucial role in the survival and infection process of *Shigella flexneri* [1,10,11]. *Shigella*’s pathogenic capability is associated with its ability to adhere, invade, multiply, and eventually kill the colonic epithelial cells [11,12]. Virulence plasmid-encoded genes, such as the type III secretion system (T3SS), *ipa* genes, the *mxi-spa* locus, and chromosomal-encoded *icsA/virG* gene, have been shown to be involved in *Shigella*’s survival, adherence, and the invasion process [12,13,14,15,16,17,18]. *S. flexneri,* while in the host gastrointestinal tract, encounters various stimuli and pressures such as acidic pH, the presence of bactericidal bile, competition against host microflora, the compulsion of an effective adherence to the epithelial cells to prevent clearance, followed by a successful invasion and evasion from the host immune system [10,11,19]. To effectively adapt to these adverse conditions in the host during the infection process, intestinal pathogens such as *S. flexneri* have developed and adapted various methods to sense and appropriately respond to external stimuli [20].

An abundant, highly conserved molecule that acts as a secondary messenger in *Shigella* and other bacteria facilitating these cellular and molecular adaptations to the host environment is Bis- (3′-5′)-cyclic dimeric GMP (c-di-GMP) [20]. The rise in c-di-GMP levels inside the bacterial cell is known to promote flagella/pili biosynthesis, motility, biofilm formation, transcription, exopolysaccharide (EPS) production, surface adherence, invasion of host cells, and overall virulence of the bacteria [21,22,23,24,25]. Inversely, a fall in c-di-GMP level can negatively affect all these above-mentioned virulence factors. This association between increased c-di-GMP levels and biofilm production and/or motility mechanisms has been extensively researched in *E. coli*, *Salmonella enterica* serovar Typhimurium, and *Pseudomonas aeruginosa* [22]. Escalated levels of c-di-GMP control biofilm-linked targets such as exopolysaccharide production, bacterial motility, expression of surface adhesin, production of secondary metabolites which eventually help the bacteria in establishing biofilms and effective stress responses, and aids in resistance to antimicrobials [21,22,23,24,25,26,27]. The increase in motility due to c-di-GMP regulation, achieved by inducing the formation of pilli/flagella/fimbriae, helps in the formation of biofilms, its dispersal, and maturation, leading to a stable three-dimensional biofilm architecture [26,27]. Additionally, c-di-GMP is involved in adherence and attachment to host surfaces which prevent mechanical clearance and further help in the effective invasion of the host [21,22,23,24]. c-di-GMP signalling controls host cell adherence and invasion, intracellular spread, cytotoxicity, regulation of immune responses, and secretion of other virulence factors [21,22,23,24]. Therefore, understanding how the levels of c-di-GMP are modulated and how this multistep signalling works in *S. flexneri* is essential for developing novel and efficacious treatments against the pathogenic bacteria.

The key protein in question for this study-YfiB, was present as a hypothetical protein in *S. flexneri* 1c strain (SFL1613/Y394), and it was predicted to be an outer membrane protein belonging to the OmpA superfamily. It is known from the earlier studies on c-di-GMP regulation of virulence in *Pseudomonas* that YfiB protein is part of the tripartite signalling system known as YfiBNR [28], responsible for modulating c-di-GMP levels as per the stress indicators detected at the periplasm [28,29,30,31]. Even in the *Shigella* genome, YfiB is always seen to present as part of the *yfiBNR* operon system. This signalling system has also been reported in other Gram-negatives such as *Klebsiella*, *E. coli*, and *Yersinia pestis* [31]. YfiB is predicted to transduce signals into a prompt escalation of intracellular c-di-GMP levels, affecting various downstream virulence factors, however, proper understanding of YfiB remains incomplete [28,29,30,31].

**Figure 1:**
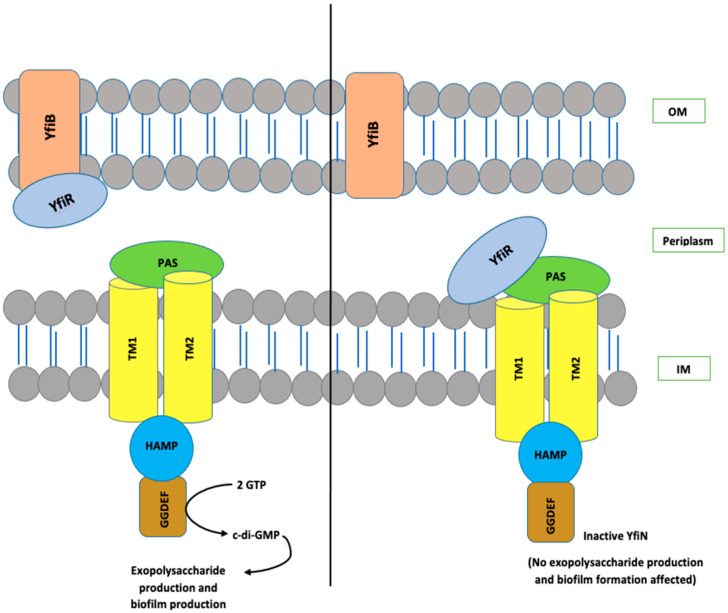
The organization and interaction model of the YfiBNR system. YfiN, an inner-membrane-located DGC, is repressed when bound to periplasmic YfiR. Whereas, dissociation of the complex by YfiB, which is a Pal-like protein located in the outer membrane, which sequesters YfiR and consequently stimulates YfiN and leads to the production of c-di-GMP. OM, outer membrane; IM, inner membrane.

The YfiBNR system consists of three different protein members: (1) an inner membrane diguanylate cyclase (DGC) responsible for the production of c-di-GMP, annotated as YfiN [28,29,30,31]; (2) an outer membrane protein, YfiB [28,29,30,31]; and (3) a periplasmic protein, YfiR, which forms an association between YfiN and YfiB [28,29,30,31]. In the studies conducted in *Pseudomonas*, it is hypothesized that YfiR allosterically inhibits YfiN by binding to its periplasmic domain and, on the other hand, YfiB positively regulates YfiN’s DGC activity. It does this by sequestering YfiR to the outer membrane, which is accomplished via membrane anchoring and peptidoglycan binding, thereby preventing its binding to YfiN and releasing its inhibition [28,29,30,31,32]. When YfiN is active, it leads to stimulation of its DGC activity, which helps in c-di-GMP production, resulting in enhanced biofilm and various other virulence factors [28,29,30,31,32]. The working model for YfiBNR functioning adapted from the *P. aeruginosa* YfiBNR system, developed by Malone, J.G. et al. (2010) [31], is illustrated in Figure 1.

In this study, we examined the functional role of YfiB in the YfiBNR system and its effect on c-di-GMP production. Regulation of c-di-GMP consequentially affects many of the virulence factors in *S.* *flexneri*, hence determining this link between YfiN and its positive regulator YfiB is essential. To this end, we generated a *yfiB* gene knockout, using double homologous recombination, and studied its effect on *Shigella*’s survival and pathogenesis, using various in vivo and in vitro virulence assays. This was followed by the in silico analysis of the protein structure, based on the previously solved crystal structure of YfiB protein from *Pseudomonas* [30]. This also aided in designing targeted mutagenesis experiments to identify critical amino acids, involved in the functioning of YfiB protein in *Shigella*. This study essentially validates YfiB’s role in regulating YfiN (DGC) activity and thereby controlling c-di-GMP levels in the bacteria, which is indispensable for *S. flexneri*’s ability to cause an effective infection in the host.

## 2. Materials and Methods

### 2.1. Strains and Growth Conditions

*S. flexneri* serotype 1c (SFL1613/Y394) is a clinical strain from Bangladesh and it was generously provided by Nils I. A. Carlin [33]. Using a sterile loop, Y394 and other *Shigella* strains were streaked from glycerol stocks and cultured on Luria Bertani (LB) agar plates and/or tryptic soy broth (TSB) agar plates with 0.01% (*w*/*v*) Congo red (CR) and were grown aerobically (180 rpm) at 30 °C in LB broth to retain the virulence plasmid. Overnight cultures were then subcultured at 1:100 dilution and grown at 37 °C with 180 rpm shaking until an optical density of 0.5 to 0.8 (log-phage culture) was obtained at 600 nm (OD_600_). The antibiotics were added as supplements, where indicated, at the following concentrations: 50 µg/mL erythromycin, 25 µg/mL chloramphenicol, 100 µg/mL ampicillin, 50 µg/mL kanamycin, and 20 µg/mL gentamicin. Additionally, 0.2% arabinose was added for all inducible plasmids. All plasmids, strains, and primers used in this study are listed in Supplementary Tables S1 and S2.

### 2.2. Knocking out yfiB Gene to Create the KO Mutant and Its Complementation

*yfiB* knockout (KO) mutant SFL2641 (ΔYfiB) was created employing the lambda red double recombination approach established by Datsenko and Wanner with a few modifications [34,35], using the helper plasmid pKD46 and pKD3 plasmid containing chloramphenicol resistance (CM) for construction of the knockout template. A PCR-based methodology was used for the construction of the knockout template. The CM gene was amplified using the miniprep DNA of pKD3 using primer pairs containing 80 bp overhangs, which were homologous to the downstream and upstream of the *yfiB* gene. *S. flexneri* 1c strain (Y394), containing the pKD46 plasmid, encoding the lambda red genes (*gam*, *beta*, and *exo*) when induced with 100 mM arabinose, was transformed with the knockout template. The positive mutants were first screened on LBA plates containing chloramphenicol and then via colony PCR followed by sequencing.

Complementation of *yfiB* KO mutant was obtained by cloning the PCR-amplified *yfiB* gene from the genome of *S. flexneri* 1c SFL2608 strain; this PCR product was then purified and digested with *Nco*I and *Hind*III. Digested PCR product encoding *yfiB* was cloned into the pBAD_*Myc*_HisA vector, which contains a C-terminal 6xHis tag fused to the protein for checking expression SFL2642 (YfiBComp).

### 2.3. Biofilm Assay

The protocol used for the biofilm assay was tailored from the method initially established by Christensen et al. with a few modifications [19,36]. A single colony of each bacterial strain was inoculated into a tube containing 10 mL of Tryptone Soy Broth (TSB). The cultures were incubated overnight at 30 °C with 180 rpm shaking. On the following day, each of the cultures was diluted to 1:100 in 5 mL into two tubes, one with glucose (1%) and bile salts (0.4%) and the other without the glucose and bile salts [19,37,38]. The OD_600_ of diluted cultures were checked to make sure the cultures were uniform. Next, 100 µL control media and diluted cultures were inoculated per well in the sterile 96-well microplates (Thermo Fisher, VIC, Australia). The plates were incubated for 6, 12, and 24 h at 37 °C statically. After incubation, the culture media were removed from the plate gently by slanting the plate and gradually removing the medium using a multichannel pipette, and 1X PBS was used to rinse the wells. The wells were left to dry for 30 min, after which methanol was used to fix the dried surface biofilm and then 0.5% crystal violet was used for staining. After staining, the wells were washed 4 times with MilliQ water, followed by drying the plate for over 3 h. To each well, 200 µL of 95% ethanol was added after drying and incubated for an hour at 4 °C to avoid the evaporation of ethanol. The absorbance of all the wells was recorded at 595 nm wavelength (OD_595_) using the Tecan plate reader. A biofilm assay with each strain was performed at least 4 times independently with three biological repeats; shown in the graph is the mean of all experiments.

### 2.4. In Vitro Bacterial Adhesion Assay

A bacterial adhesion assay was carried out using Baby Hamster Kidney fibroblast (BHK) cells [39,40,41]. *Shigella* strains were grown in TSB–sodium deoxycholate medium until an OD_600_ = 0.6–0.7 (late log phase) was obtained and appropriately diluted in normal saline to a required 2 × 10^9^ CFU/mL concentration for infection. This prepared culture was used to infect the BHK cells (100 uL/each well), then incubated at 37 °C for 2–3 h for the infection process. After incubation, the cells were rinsed 4 times using 1× PBS, to eliminate all non-adhered bacteria, and were lysed using 1% Triton-X by incubating for 10 min at room temperature on a shaker. LB medium was added to the lysed cells to recover the adhered and invading bacteria. Serial dilutions were prepared of the suspension, plated on LBA, and the plates were incubated at 37 °C, overnight. After incubation, the number of colonies on the plates were counted and the total colony-forming unit (CFU) was determined., counting of colonies was performed, as well as calculating the total colony-forming unit (CFU). The percentage of adhered bacteria was estimated by dividing the total CFU of adhered bacteria by the total CFU of the inoculum [39].

### 2.5. In Vitro Invasion Assay and Microscopy

The bacterial invasion assays [40,42,43] were performed using an epithelial cell line, Baby Hamster Kidney fibroblasts (BHK) cells. Cells were grown in tissue culture flasks (25 cm^2^) to 80% confluency. The incubation of the cells was performed using a CO_2_ incubator with 5% CO_2_ at 37 °C, using the tissue culture media Dulbecco’s Modified Eagle’s Medium (DMEM) supplemented with 10% (*v*/*v*) Fetal Bovine Serum (FBS), 2 mM glutamine, and 1X non-essential amino acids. The bacterial culture was prepared for invasion assay by taking a single isolated colony from each of the *S. flexneri* strains that need to be tested, these were cultured in 5 mL of LB with suitable antibiotics. Cultures were grown overnight at 30 °C to preserve the virulence plasmid. The following day, the cultures were diluted 1:100 in LB with 0.1% deoxycholate, containing appropriate antibiotics, and allowed to grow until OD_600_ = 0.6–0.8 (mid-log phase) was obtained at 37 °C. The appropriate number of bacteria were harvested via centrifugation and resuspended in 1 mL of 1× PBS to obtain the culture of 2 × 10^9^ CFU/mL. From this culture, 0.1 mL was used per well of a 6-well tissue culture plate to infect the BHK cells. Centrifugation of the plates for 10 min at 1000× *g* was done and then incubated for 30 min at 37 °C in 5% CO_2_ level. The media were then removed and wells were washed four times with 1 mL of 1× PBS. The tissue culture media containing gentamycin (10 µg/mL) were added, and the plate was incubated for another 60–90 min (2 h maximum, including 30 min above) at 37 °C and 5% CO_2_.

Staining and Data collection: After incubation, the media were aspirated and wells were rinsed with 1 mL of 1× PBS two times. Next, 0.5 mL of filtered Wright–Giemsa stain was added to each well for staining the cells. After 2 min, the stain was washed two times with 2 mL MilliQ water, and the plates were inverted for drying. The invasion of bacteria per BHK cells was determined by enumeration of at least 300 BHK cells [40] and the total bacteria present using a 100× oil immersion microscope; this process was performed in duplicates with 3 repeats.

### 2.6. In Vitro Plaque Assay

Plaque assay was carried out using the HeLa cell line [44,45]. *Shigella* strains were grown in TSB medium until OD600 = 0.4–0.5 (mid-log phase) was obtained, and appropriately diluted in normal saline to a required 1 × 10^7^ CFU/mL concentration. This prepared culture was further diluted to 10^6^ to 10^4^ CFU/mL to infect the HeLa cells (100 uL/each well), and subsequently, the plates were incubated at 37 °C, 45–90 min for the infection process. The medium was removed after incubation, cells were washed twice with 1× PBS, and 2× gentamycin medium was added to each well. Gentamycin medium was used to remove all external bacteria, which results from futile spreading into the extracellular medium, rather than the neighbouring cells. This ensures that each plaque formed, parallels to a sole invasion occurrence at the start of the experiment [43,44,45]. The plates were then re-incubated at 37 °C for 48–72 h, plaques were mostly visible after 48 h. When the plaques are visible, the plate was taken out and washed with 1× PBS to wash away dead cells as they detach from the plate surface. Wright–Giemsa stain was used to counterstain the intact monolayer to make the plaques evident (clear spaces) against the stained background [44,45]. The overall number of plaques was counted and the mean number of plaques per bacteria was plotted on the graph. The experiment was carried out for 50 technical repeats with 2 biological repeats each time. A few of the stained plaques were also observed under the microscope to see how the plaques looked under 40× magnification and whether there were any observable morphological changes of the cells near and around the plaques.

### 2.7. In Vivo Bacterial Colonization Assay Using Caenorhabditis elegans

The bacterial colonization assay was executed using the *C. elegans* N2 strain (WT). These nematodes were cultured on a modified nematode growth medium (mNGM), plates already containing *E. coli* OP50 grown on it [46,47,48]. L4-stage *C. elegans* of a synchronized population was collected off these *E. coli* OP50 plates using S-basal buffer, and it was subjected for 3 h to 200 µg/mL of gentamycin to remove any *E. coli* OP50 cells present on the surface of the worms. Then, the worms were meticulously rinsed with S-basal buffer to eliminate any remaining antibiotics. Bacterial strains were grown at 37 °C overnight on an mNGM plate to prompt the expression of genes encoded by the virulence plasmid. The washed worms were put onto these plates with the seeded *Shigella* strains and incubated for 24 h at 22 °C. The next day, 15 worms were picked from each plate, and to anesthetize these worms, they were rinsed meticulously using S-basal containing 1 mM sodium azide. The worms were subjected for 3 h to 200 µg/mL of gentamycin to remove any bacterial cells present on the surface of the worms. After 3 h, the worms were rinsed three times with S-basal buffer containing 1 mM sodium azide. The washed worms were resuspended in S-basal buffer containing 0.1% Triton-X and were lysed mechanically using sterile glass beads. The obtained lysate was serially diluted using 1X PBS, and appropriate dilutions were prepared. These dilutions were then plated on LB agar plates, containing the suitable antibiotics, to obtain the intraluminal bacterial counts. These plates were incubated at 37 °C for 16 h and the number of bacteria per 15 nematodes was determined. The analysis was based on three independent repeats.

### 2.8. In-Silico Analysis of YfiB Protein

YfiB protein is present as a hypothetical protein in the *Shigella* genome, therefore, the 5-step identification system for a hypothetical protein was applied [49]. Functional analysis by identifying the conserved domain was conducted using NCBI-protein BLAST [50] and conserved domain database [51], followed by analysing its physicochemical parameter using Expasy’s ProtParam tool [52]; determining the subcellular localization using CELLO [53], PSORTb [54], and PSLpred [55]; the presence of transmembrane helices using TMHMM [56] and HMMTOP [57] tools; and lastly, determining whether they are concerned in virulence of *S. flexneri* using VICMpred [58] and VirulentPred [59].

The protein sequence of *Shigella* Y394′s YfiB was analysed and compared to *P. aeruginosa*’s YfiB. The similarity between the protein sequences and the conserved amino acid residues was evaluated using NCBI-protein BLAST and Clustal-omega sequence alignment tool [60]. The I-TASSER (Iterative Threading ASSEmbly Refinement) tool [61,62] was utilized to predict the three-dimensional structure of YfiB protein of Y394, the already solved crystal structure of *Pseudomonas* YfiB protein [29,30] was used as a template to determine the 3D structure. Significant amino acids of YfiB protein known from previous studies in *Pseudomonas* [29,30] were identified, and based on this analysis, targets for mutagenesis in Y394 YfiB protein were selected.

YfiB homologs were identified in various other Gram-negative species, protein homologs (E-value < 10^−4^) were aligned using Clustal Omega, and conserved amino acid residues were determined. Sequence conservation at each position was evaluated using the WebLogo tool [63], it creates a stack of symbols, the altitude of the pile at each position signifies the sequence conservation and the height of the symbols denotes the comparative incidence of individual amino acids at that locus [63]. Phylogenetic analysis was also carried out of the YfiB homologs, and an illustrative tree based on the multiple sequence alignment of YfiB homolog protein sequences was generated using the MEGA software [64], the Maximum Composite Likelihood technique was used to determine the evolutionary distances [64,65,66]. UniProt accession numbers of all YfiB protein sequences used for the in silico analysis are listed in Supplementary Table S3.

### 2.9. Site-Directed Mutagenesis and Protein Expression

For the targeted mutagenesis experiment, four amino acid targets were selected, these being Cys19Gln20 mutated to Ala19Glu20; Pro22Gln23 mutated to Ala22Glu23; Glu29Gln30 mutated to Ala29Glu30, which are part of the YfiB peptidoglycan linker sequence, and Ser36 mutated to Ala36. Site-directed mutants were synthesized by GenScript USA, cloned into the expression pBAD_Myc_HisA vector, using the *BamH*I and *Apa*I sites. For inspecting the expression of the YfiB protein, *Shigella* strains, containing the pBAD_Myc_HisA_YfiB vector, were grown in LB broth medium with 100 µg/mL ampicillin and 50 µg/mL erythromycin at 37 °C. Once the OD_600_ of the cultures reached 0.6, 1 mL of the cell culture was pelleted down and suspended in an appropriate volume of protein loading dye containing β-mercaptoethanol as the reducing agent to obtain an equal concentration of proteins in each sample. This protein suspension was boiled at 100 °C for 5 min before loading 10 µls of it into the wells of two SDS gels. Coomassie blue staining of one loaded SDS gel was performed to check for equal total protein loading and Western transfer with blotting was performed with the second gel using the anti-HisA [67] antibody to check for protein expression (Supplementary Figure S3C,D).

### 2.10. Quantification of c-di-GMP

The levels of c-di-GMP produced in the different *Shigella* strains was estimated using the Cyclic-di-GMP ELISA kit by Cayman Chemicals, MI, USA. This ELISA kit is based on the principle of competition between the native c-di-GMP, and a c-di-GMP-horseradish peroxidase conjugate (c-di-GMP tracer) provided in the kit. Cell lysates of the *S. flexneri* strains: SFL2608 (1c WT), SFL2641/ΔYfiB, SFL2642/YfiBComp, along with the YfiB site-directed mutant strains—SFL2645 (Cys19Gln20->Ala19Glu20), SFL2646 (Pro22Gln23->Ala22Glu23), SFL2647 (Glu29Gln30->Ala29Glu30), and SFL2648 (Ser36->Ala36), and the empty pBAD_Myc_HisA vector strain (SFL2650), were prepared in Thermo Fisher’s Bacterial Protein Extraction Reagent (B-PER^TM^). The competitive ELISA assay and calculation of c-di-GMP concentration in each strain were performed according to the instructions provided in the kit’s manual. The assay for each strain sample was performed in duplicates and three independent repeats were carried out for final quantification of native c-di-GMP levels based on the c-di-GMP standard curve.

### 2.11. Statistical Analysis

Statistical analysis to evaluate the significance in each experiment, which was based on at least 3 or more trials, was performed using GraphPad Prism’s unpaired Student’s *t*-test (standard cut-off for significance: *p* < 0.05).

## 3. Results

### 3.1. Comparing the c-di-GMP Content of the Wild-Type and Mutant Strain

**Figure 2:**
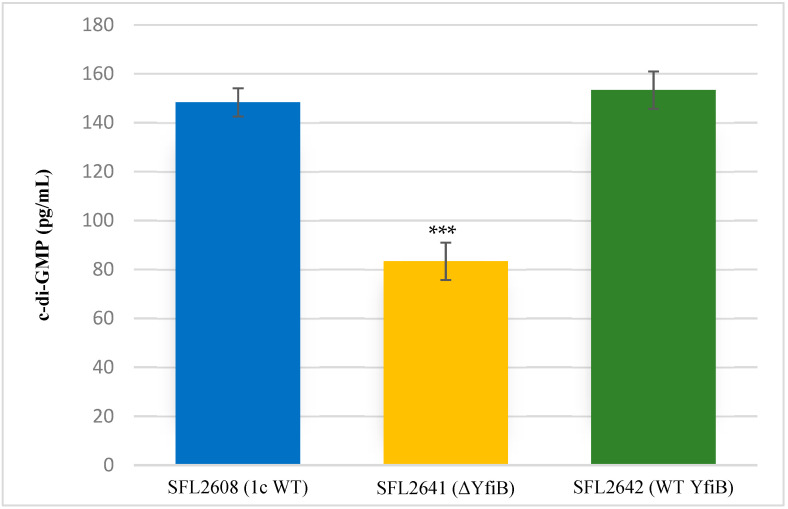
Quantification of intracellular c-di-GMP levels. Bar graph showing the levels of native c-di-GMP for *S. flexneri* strains: 1c wild-type (SFL2608), yfiB KO mutant SFL2641 (ΔYfiB), yfiB-complemented strain SFL2642 (YfiBComp). The c-di-GMP concentration was analysed using a Cyclic- di-GMP ELISA kit by Cayman Chemicals. The calculations are based on a standard curve for c-di-GMP and assaying each sample in duplicates. Statistically significant variance is denoted by asterisks (*** = *p* value < 0.001). The standard deviation of data from three autonomous experiments is depicted by the error bars.

Quantification of c-di-GMP was performed to examine whether the KO of yfiB gene leads to the inactivation of yfiN gene responsible for c-di-GMP production. According to the YfiBNR working model (Figure 1), deleting the positive regulator YfiB, leads to YfiR being permanently bound to YfiN, inactivating it in this process and hence resulting in reduced intracellular levels of c-di-GMP [31,32]. To test this working hypothesis, the concentration of native c-di-GMP present in each *Shigella* strain used in this study was quantified using a c-di-GMP ELISA kit. It was observed that the KO mutant SFL2641 (ΔYfiB) strain had a significantly reduced amount of c-di-GMP, about 83.3 pg/mL in comparison to 148.3 pg/mL of c-di-GMP in 1c wild-type strain (SFL2608). The complement strain SFL2642 (YfiBComp) showed a 153 pg/mL concentration of native c-di-GMP, clearly indicating that the wild-type phenotype is restored when yfiB is added back via cloning (Figure 2).

### 3.2. Biofilm Formation Is Significantly Slower in the yfiB-Deleted Shigella Strain

Enteric pathogens such as *Shigella* produce biofilms to enhance survival and protect themselves from harsh environments such as the bactericidal bile present in the human GIT [19,37]. Biofilms are defined as structured groups of bacteria entrenched in a self-constructed matrix that is made up of various proteins, extracellular DNA, and exopolysaccharides [19,37]. To test the effect of altered c-di-GMP levels in *Shigella* and to assess whether the YfiB protein along with the YfiBNR system plays a role in biofilm formation, we knocked out the *yfiB* gene from *S. flexneri* 1c strain (SFL2608) and measured the extent of biofilm produced by the wild-type and mutant strain SFL2641 (ΔYfiB) at 6, 12, and 24 h in presence and lack of 0.4% bile salts and 1% glucose [19,37]. The *yfiB* gene was also complemented in the knockout strain SFL2642 (YfiBComp) by cloning the gene in the pBAD_*Myc*_HisA vector and introducing this plasmid in the SFL2641 strain, to see whether the wild-type function is reinstated.

**Figure 3:**
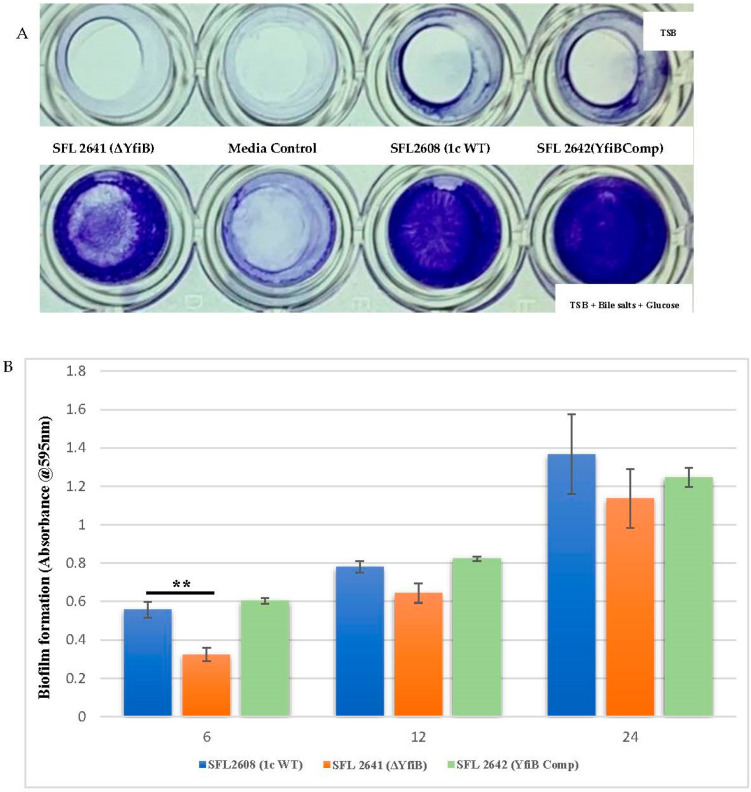
Quantification of biofilm formation by *S. flexneri* strains. (**A**) Examination of biofilm formation in plate wells when grown with and without bile salts in TSB media at 6 h. Biofilm formation was performed in a 96-well plate, the 1c WT (SFL2608), *yfiB* KO mutant SFL2641 (ΔYfiB) and *yfiB*-complemented strain SFL2642 (YfiBComp) were cultured in Tryptone Soy Broth (TSB) medium with and without bile salts and glucose for 6, 12, and 24 h static. The observed biofilm was stained using crystal violet and assessed by determining the optical density at 595 nm (OD595). (**B**) Biofilm formation of 1c WT (SFL2608), *yfiB* KO mutant SFL2641 (ΔYfiB), and *yfiB*-complemented strain SFL2642 (YfiBComp) at 6, 12, and 24 h; at 6 h, considerable difference was seen between the WT strain and *yfiB* KO mutant. The Y-axis represents the biofilm amount relative to the blank media. A Student’s t-test was conducted for pairwise comparison of the biofilm formation and the analysis was based on three independent biological repeats, with each assay undergoing at least four technical repeats. The two asterisks represent the variation observed being statistically significant (*p* < 0.01) and error bars depict the standard deviation of data based on the three independent experiments.

As seen in Figure 3A, biofilm formation remained minimal in the lack of bile salts and glucose, in comparison with a substantial increase of biofilm formation when bile salts and glucose are present. Significantly slower production of biofilm was observed in the *yfiB* KO mutant SFL2641 (ΔYfiB) when compared to the 1c WT (SFL2608) strain. The biofilm produced by SFL2641 was considerably affected at 6 h of incubation, but it showed no difference to the WT biofilm production at 12 and 24 h. The complemented strain (SFL2642) showed similar amounts of biofilm production as WT and, therefore, it could be concluded that complementing the *yfiB* gene in the mutant strain reinstates the wild-type function (Figure 3B).

### 3.3. yfiB-Deleted Shigella Strain Shows a Decreased Adhesion and Invasion of BHK Cells and Displays an Attenuated Ability to Form Plaques in HeLa Cells

Infiltrating the colonic and rectal epithelium, proliferating intracellularly, spreading from cell to cell, are the three fundamental features of all virulent *S. flexneri* strains [68], which characteristically leads to severe host tissue damage and other shigellosis-associated manifestations [1]. Various attachment and internalization mechanisms are required by *Shigella* for the invasion of mammalian cells, with the bacteria eventually residing in the host cell cytoplasm [11,12,69]. After the invasion, the intercellular spread can occur in two ways: (1) lysis of the infected cells and discharge of intracellular bacteria; or (2) individual bacteria evading the initially infected cell before lysis and attacking the neighbouring cells without being exposed to the outside environment [44]. Even in the second case, the initially infected cell is killed or lysed by the intracellular bacteria [44]. This kind of interaction proposes that an individual *Shigella*-infected host cell would lead to an advanced infection of the surrounding cells and create a region of dying or dead host cells, which is also known as a plaque [44,45]. To determine the set of virulence genes responsible for the adhesion, invasion, and intercellular spread in host cells, in vitro assays with cultured epithelial cell monolayers, such as HeLa and BHK cell lines, have been extensively used, which mimics the in vivo infection model [40].

**Figure 4:**
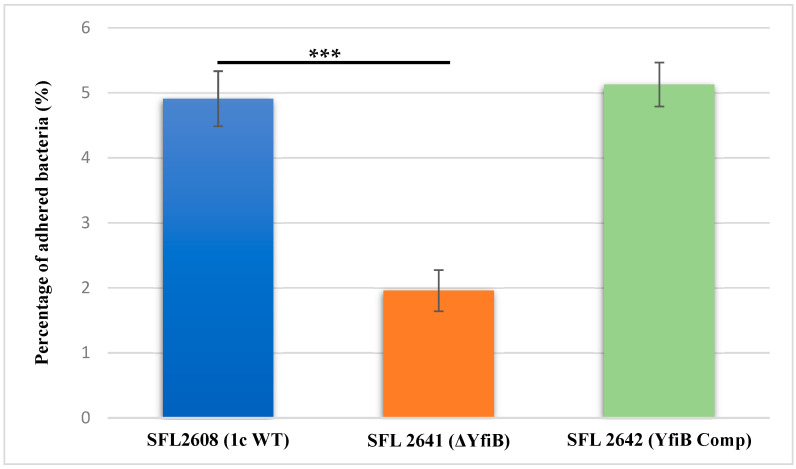
Graphical representation of percentage adhesion to BHK cells by *S. flexneri* strains. The 1c (SFL2608), *yfiB* KO mutant SFL2641 (ΔYfiB), and *yfiB*-complemented strain SFL2642 (YfiBComp) were used to infect the BHK cells. After infection, BHK cells were washed and lysed; lysates were serially diluted before plating on an LB agar plate for enumeration and colony-forming unit (CFU) calculation. The percentage of adhered bacteria was calculated by dividing the total CFU of adhered bacteria by the total CFU of the inoculum. The Y-axis represents the percentage of adhered bacteria to BHK cells after 2 h post infection at 37 °C/5% CO_2_. The results were obtained from six autonomous experiments, and to analyze the statistical differences in the adhesion, a Student’s *t*-test was performed. Three asterisks represent that the variance observed were statistically significant (*p* < 0.001) and standard deviation of the data from the six autonomous experiments is depicted by the error bars.

To investigate the involvement of the *yfiB* gene and *yfiBNR* operon in the initial attachment or adherence of bacteria to the host cell, an in vitro adhesion assay was performed, in which we infected BHK monolayers with the following *Shigella* strains: 1c WT (SFL2608), *yfiB* KO mutant (SFL2641/ΔYfiB), and *yfiB* complement strain SFL2642 (YfiBComp). The percentage of adhered bacteria for the WT strain (SFL2608) was seen to be 2.5 times higher than the *yfiB* KO mutant (SFL2641/ΔYfiB). Statistical analysis via an unpaired Student’s t-test confirmed that the *yfiB* KO mutant had a considerably decreased capacity to adhere to the epithelial cells of the host when paralleled with the wild type (Figure 4). When the *yfiB* gene was complemented SFL2642 (YfiBComp), it was seen that the wild-type adherence ability was restored. This clearly shows the role of YfiB and YfiBNR system in *Shigella* virulence, as the very first step of its infection process is adherence to host cells.

**Figure 5:**
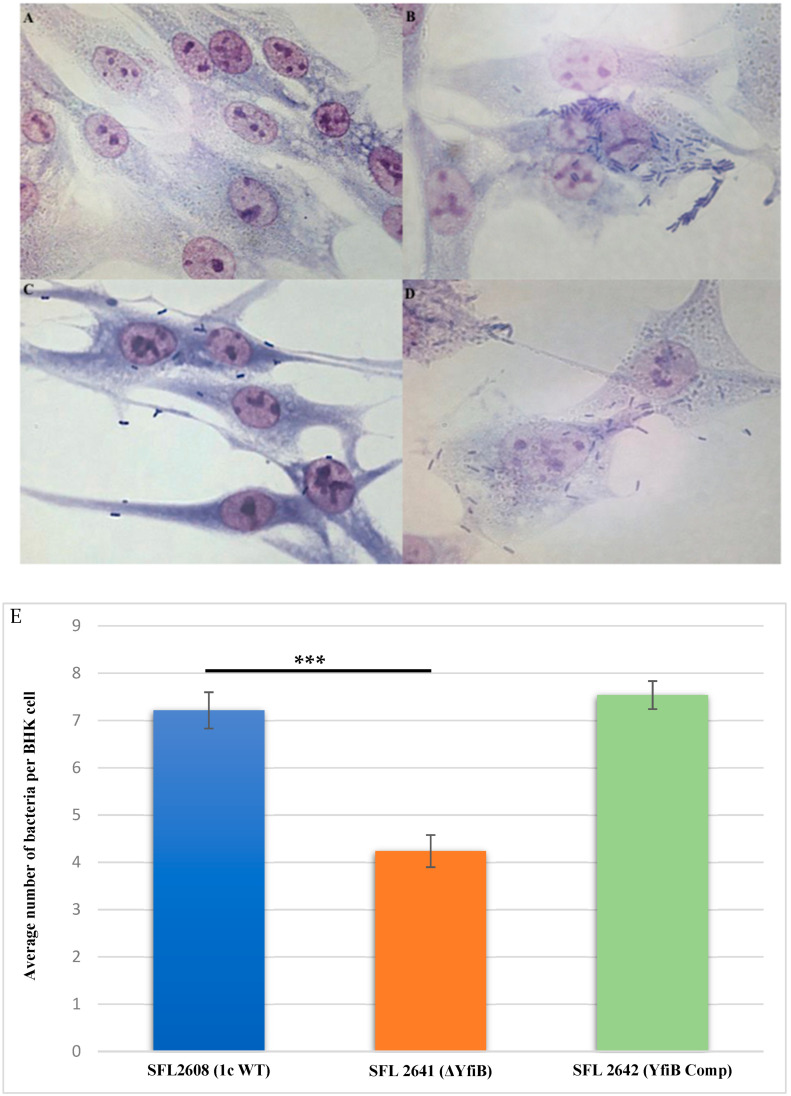
Invasion of BHK cell monolayers by *S. flexneri* strains. Microscopic images of infected BHK cells, after 2 h post infection at 37 °C and at 5% CO_2_; Giemsa stain was used to stain the monolayers, and the plates were observed under the microscope. (**A**) Microscopic image of noninfected BHK cells under 100× oil immersion. (**B**) Microscopic image of BHK cells infected with the 1c WT (SFL2608) under 100× oil immersion. (**C**) Microscopic image of BHK cells infected with *yfiB* KO mutant SFL2641 (ΔYfiB) under 100× oil immersion. (**D**) Microscopic image of BHK cells infected with *yfiB*-complemented strain SFL2642 (YfiBComp) under 100× oil immersion. (**E**) *Shigella* 1c WT (SFL2608), *yfiB* KO mutant SFL2641 (ΔYfiB), and *yfiB*-complemented strain SFL2642 (YfiBComp) were used to infect the BHK cells. The Y-axis represents the number of internalized bacteria per BHK cell after 2 h post-infection at 37 °C/5% CO_2_. The results were obtained from three independent experiments and were derived from scoring at least 300 BHK cells. To analyze the statistical differences in the invasion, a Student’s *t*-test was performed. Three asterisks represent that the variance observed was statistically significant (*p* < 0.001) and standard deviation of data from three autonomous experiments is depicted by the error bars.

Examining the consequence of deleting the *yfiB* gene and disrupting the *yfiBNR* operon on the invasive potential of *S. flexneri* serotype 1c was performed by carrying out an in vitro invasion assay, in which BHK cells were infected with the following strains: 1c WT (SFL2608), *yfiB* KO mutant (SFL2641/ΔYfiB), and *yfiB* complement strain SFL2642 (YfiBComp). The bacteria invading the BHK monolayers were stained and counted by microscopy, the average number of bacteria invading per BHK cell was found to be around two folds greater in wild type when compared with the *yfiB* KO mutant (Figure 5A–D). Statistical analysis confirmed that the *yfiB* KO mutant had a considerably reduced capability to invade host epithelial cells when paralleled with the wild-type strain. When the *yfiB* gene was complemented, it was seen that the wild-type invasion ability was restored, which indicates the role of YfiB and YfiBNR system in the invasive potential of *S. flexneri* serotype 1c (Figure 5E).

**Figure 6:**
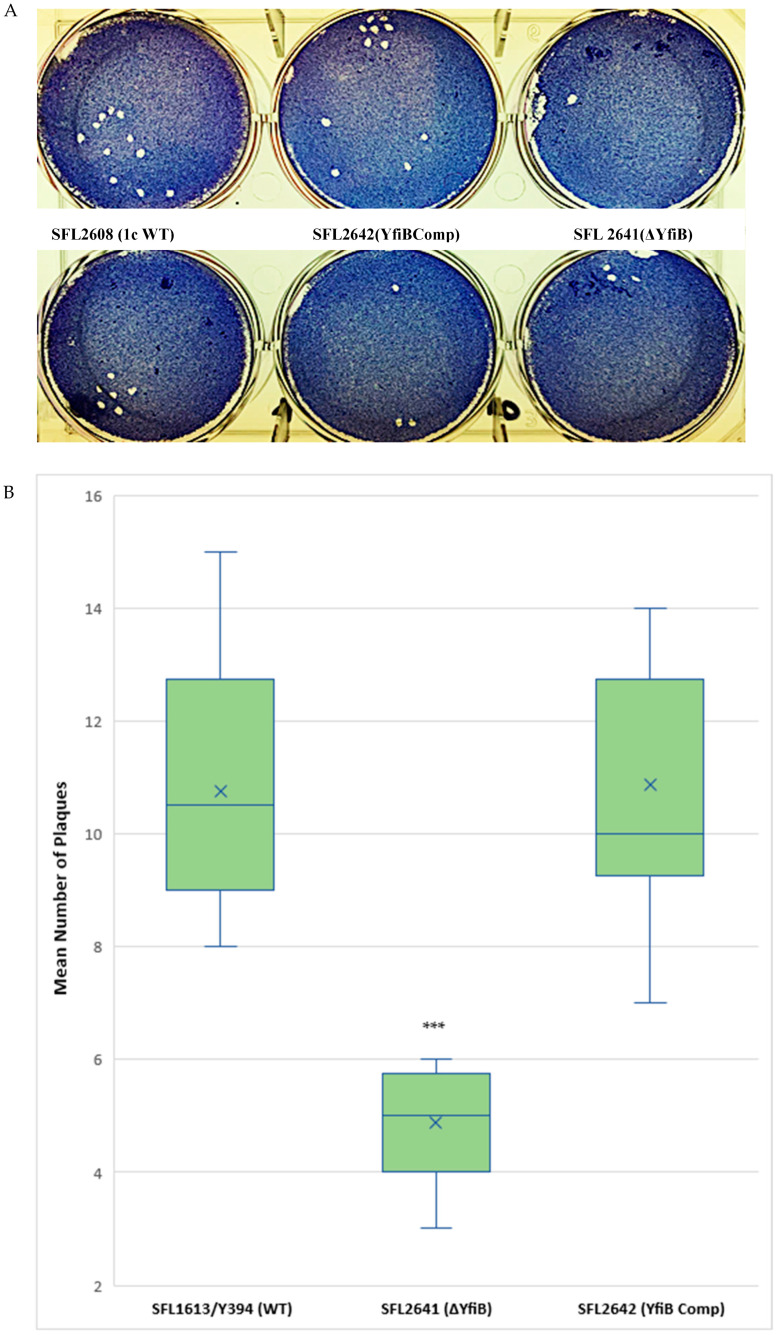
Plaque formation in HeLa cells by *S. flexneri* strains. (**A**) Visual assessment of the plaques formed on the cellular monolayer at 72 h post infection, with clear plaques representing dead cells in a 6-well plate with HeLa monolayer stained with Giemsa. (**B**) Boxplot showing the mean number of plaques formed by each strain: 1c WT (SFL2608), *yfiB* KO mutant SFL2641 (ΔYfiB), and *yfiB*-complemented strain SFL2642 (YfiBComp). Plaque assay was performed in 6-well plates by infecting confluent HeLa cell monolayers with *Shigella* strains. Infection was carried out for 48–72 h, post-infection, the monolayer was washed and stained with Giemsa, resulting in clear plaques that are visible against the coloured background. The number of plaques formed were counted for each strain and then plotted. The results were obtained from 50 independent technical repeats with two biological repeats each time. To analyse the statistical variances in plaque formation between the strains, a Student’s *t*-test was performed and statistically significant (*p* < 0.001) variations are denoted by the three asterisks.

A plaque assay was also performed using HeLa cells to understand the two vital steps involved in the *Shigella* infection process—invasion and intracellular spread of the bacteria [44]. The number of plaques evaluates the invasive ability of the bacteria, and the size dimensions of the plaques reveals the bacteria’s capability to spread within an epithelium [44,45]. We infected HeLa cells with *Shigella* strains—1c WT (SFL2608), *yfiB* KO mutant (SFL2641/ΔYfiB), and *yfiB* complement strain SFL2642 (YfiBComp), for 48–72 h. It was observed that the number of plaques produced by the *yfiB* KO mutant SFL2641 (ΔYfiB) was 2.5 times lesser than the 1c WT strain (SFL2608). When the *yfiB* gene was complemented SFL2642 (YfiBComp), it was seen that the wild-type plaque-forming ability was restored. However, there was no substantial difference observed in the diameters of plaques formed between the strains (Figure 6A,B). A small number of plaques seen in the *yfiB* KO mutant indicates its inability to adhere to the host cells and disseminate from the initial site of infection, thereby showing significantly less invasion capability compared with the 1c WT strain.

### 3.4. yfiB KO Influences C. elegans Lifespan and Survival during Infection

*Shigella* has a very narrow host range, infecting only primates and humans [1]. The most used in vivo animal models are the murine pulmonary and guinea pig keratoconjunctivitis models [46]. Although these animal models have been used for years, they lack clinical relevance of infection site and the symptoms produced when compared to a *S. flexneri* infection in humans [46]. *C. elegans*, which is a soil-dwelling nematode, has recently been used for several enteric pathogens to study host–pathogen interactions [47]. *C. elegans* show morphological resemblances with the human intestinal cells and significant similarities with the human innate immune system, making it a good animal model to study enteric bacterial infections [46,47,48]. It was first revealed from the studies conducted by Burton et al. that there is an accumulation of virulent strains of *S. flexneri* in the *C. elegans* guts, affecting the lifespan of the worms, however, the avirulent strains tend to get digested [48]. Bacterial accumulation of pathogenic bacteria in the worm’s intestine can drastically shorten the lifespan of worms and, on the other hand, accumulation of nonpathogenic *E. coli*, such as OP50, which is ingested as feed increases the lifespan of worms [46,47,48]. Thus, bacterial accumulation assay using *C. elegans* assisted us in evaluating the role of *yfiB* gene, the *yfiBNR* operon system, and modulation of c-di-GMP in *Shigella*’s infection of the nematodes and evading its immune defense system.

**Figure 7:**
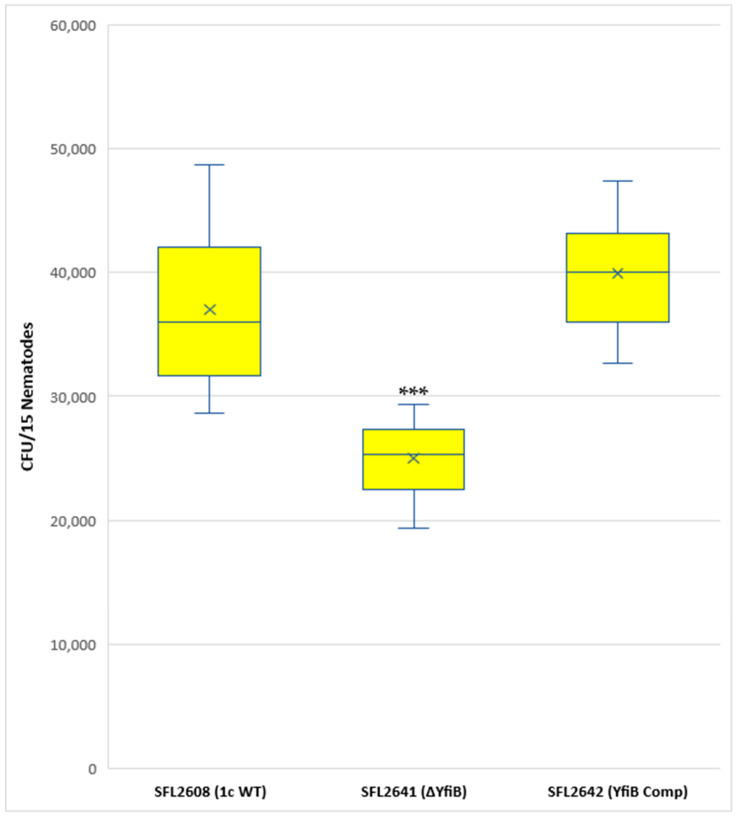
Quantification of bacterial colonization in *C. elegans*. The synchronized L4-stage N2 nematodes were fed with 1c WT (SFL2608), *yfiB* KO mutant SFL2641 (ΔyfiB), and *yfiB*-complemented strain SFL2642 (YfiBComp) for 24 h at 22 °C. From each plate, 15 worms were picked, lysed utilizing glass beads, after which suitable dilutions of individual lysate were plated on LB agar plates to attain bacterial colonization numbers. To analyse the observed variance in colonization numbers between the strains, a Student’s t-test was performed. The 1c WT (SFL2608) and *yfiB*-complemented strain SFL2642 (YfiBComp) showed higher accumulation in *C. elegans*, when compared to *yfiB* KO mutant SFL2641 (ΔyfiB) strain. Statistically significant (*p* value < 0.001) variance is indicated by the three asterisks and the analysis was based on three independent repeats.

A synchronized population of L4 larva stage hermaphrodite N2 nematodes was prepared and fed with 1c WT (SFL2608), *yfiB* KO mutant SFL2641 (ΔyfiB), and the *yfiB*-complemented strain SFL2642 (YfiBComp). Colonization numbers of the wild-type strain were around two folds greater (average CFU/worm) than those of the mutant (average CFU/worm for SFL2641 (ΔyfiB). The average CFU/worm for the complemented strain SFL2642 (YfibComp) was similar to that of the wild type, indicating that the invading ability of the wild-type strain was restored. Statistical analysis confirmed that the accumulation in the *C. elegans* intestinal lumen by the wild-type strain was considerably higher compared with the *yfiB* KO mutant (Figure 7).

### 3.5. In Silico Investigation of the YfiB Protein Elucidates its Structure, Function, and Conserved Nature across Bacterial Species

YfiB was present as a hypothetical protein in the *S. flexneri* 1c strain (Y394) genome, in silico functional analysis was carried out using NCBI-protein BLAST and the conserved domain database to identify homologs across other bacteria and conserved domains. Homologs across other Gram-negative bacterial species were found. YfiB is a 17.2 kDa putative lipoprotein or an integral membrane protein, with an OmpA domain and Omp_C-like superfamily domain (Supplementary Figure S1A). Functional prediction of YfiB as per the conserved domains showed that it might be involved in transmembrane transporter activity; in cell growth and adherence; in response to stress and/or external encapsulating structure. Physicochemical properties, subcellular location, presence of transmembrane domains, and involvement in virulence were also studied, using various bioinformatics tools listed in the methods section. YfiB was predicted to be located in the outer membrane, comprising of a PAL-like peptidoglycan (PG) domain and no transmembrane helices. Consensus prediction of gene ontology (GO) showed that YfiB was involved in molecular and biological processes of the bacteria and the protein had a 0.271 grand average of hydropathicity (GRAVY).

The YfiBNR system has been previously studied in *Pseudomonas* [31,32], and protein crystal structures of all the three proteins involved in the operon have been solved [28,29,30,31,32]. When the YfiB protein sequence of *Shigella* was compared to that of *Pseudomonas* via NCBI nucleotide and protein BLAST, no considerable similarity was found between the nucleotide or protein sequences as per the default BLAST settings. The protein sequences of YfiB from *Shigella* and *Pseudomonas* were aligned using the ClustaW tool to determine the conserved amino acid residues between the two proteins. It was observed that the crucial amino acid residues were conserved between the two proteins, as seen in Supplementary Figure S1B. The amino acids involved in the PAL (peptidoglycan associate lipoprotein) domain which aid in proper binding to the peptidoglycan are conserved in both, these being asparagine (N68), aspartic acid (D102), and glycine (G105) [31,32]. There is also the presence of an N-terminal, 13-amino-acid-long, linker sequence containing conserved critical residues. This linker sequence was predicted to be involved in binding to PAL domain, signalling conducted by YfiB, outer membrane sequestration of YfiR, and it affects biofilm formation and adhesion of bacteria [28,29,30,31,32]. An important amino acid cluster from position 35 to 55 is present in the YfiB protein which is involved in the activation of YfiB and plays a role in appropriate YfiR sequestration by YfiB in the periplasm [28,29,30,31,32]. Identifying these important amino acids helped in selecting the targets for the following site-directed mutagenesis experiments in the YfiB protein of *S. flexneri*.

To model the 3D structure of *S. flexneri’*s YfiB protein, the I-TASSER tool was used, which predicts protein structure and function based on protein sequence, it identifies structural templates from the database by the multiple threading method known as LOMETS [61,62]. Functional understanding of the target sequence is then attained by rethreading the 3D models using BioLiP, which is a protein functional database [61,62]. To establish the structure of *Shigella* YfiB protein, I-TASSER utilized the solved crystal structure of YfiB protein from *Pseudomonas* [29,30]. It was found that the YfiB protein is composed of a core OmpA-like domain (27–160 amino acids), has an N-terminal signal peptide (1–26 amino acids), and comprised α1-3 (three alpha-helices) and a β1–β4–β2–β3 network (anti-parallel β-sheet topology) (Supplementary Figure S1C).

It was observed that the presence of YfiB is quite widespread, its homologs (E-value < 10^−4^) in other Gram-negative pathogens were identified and selected for determining the sequence conservation at each position and phylogenetic evaluation. Conservation of amino acids at each position of the protein was analysed using the WebLogo tool, all the YfiB homolog sequences were first aligned using Clustal Omega (Supplementary Figure S2A), and then a sequence logo was created. Supplementary Figure S2B shows the graphical representation of all amino acids, the altitude of the pile at each position indicates sequence conservation and the height of the characters describes the comparative frequency of individual amino acids at that locus [63]. It was seen that the majority of the amino acid residues are conserved in the YfiB homologs from various Gram-negative bacteria. Using the same ClustalW alignment of the homologs, an illustrative phylogenetic tree was created using the MEGA software [64], showing the evolutionary distance between the YfiB protein homologs from different Gram-negative bacteria, calculated by the Maximum Composite Likelihood method [65,66] (Supplementary Figure S2C).

### 3.6. Mutational Analysis of YfiB Protein-Biofilm Formation and Bacterial Adhesion Assays

To determine whether some of the conserved amino acid residues of YfiB are involved in the proper functioning of the protein, targeted mutagenesis was carried out. The targets were selected based on previous studies in *Pseudomonas* YfiBNR proteins and the in silico analysis of YfiB protein in *S. flexneri* [29,30]. It was found that the 13-amino-acid-long linker sequence was the most critical for YfiB functioning, as it helps in binding to the PAL domain, YfiB signalling, and sequestering the YfiR protein. In *Shigella*, the linker sequence is only 12 amino acids long, with the majority of amino acids being conserved. Three pairs of conserved amino acid residue from the linker sequence were selected as targets, these being Cys19Gln20 mutated to Ala19Glu20; Pro22Gln23 mutated to Ala22Glu23; Glu29Gln30 mutated to Ala29Glu30 shown in Supplementary Figure S3A. Another conserved amino acid serine at position 36, which is part of an important amino acid cluster (35–55) involved in the activation and functioning of YfiB, was selected as a target and was mutated to alanine, as shown in Supplementary Figure S3B.

YfiB mutants were cloned into the pBAD_*Myc*_HisA plasmid and the protein expression of these mutants was confirmed using Western blot via an anti-HisA antibody to check whether the amino acid mutations prevented the YfiB protein from being expressed properly. YfiB is a 17.2 kDa protein and the protein bands are observed slightly above the 15 kDa band of the protein ladder. All YfiB mutants were expressed appropriately after ensuring equal total protein loading when compared to the WT YfiB protein SFL2642 (YfiBComp), as seen in Supplementary Figure S3C,D. Finally, to examine the effect of these mutations, native c-di-GMP levels were determined and biofilm formation assay, along with bacterial adhesion assay, were chosen as the functional tests to see whether the amino acid mutation caused alterations in YfiB activity and to the overall surface attachment and biofilm formation of the bacteria.

**Figure 8:**
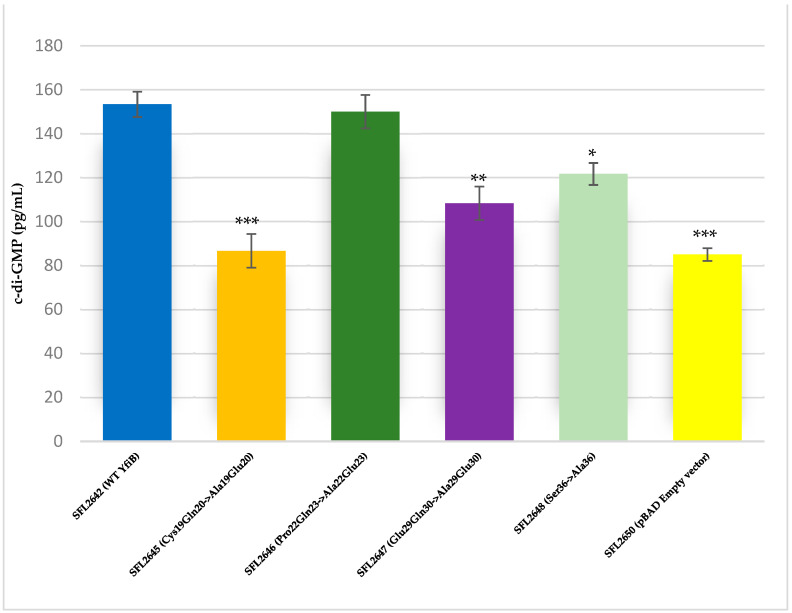
Quantification of intracellular c-di-GMP levels. Bar graph showing the levels of native c-di-GMP for *S. flexneri* strains containing WT YfiB (SFL2642), the YfiB site-directed mutants: SFL2645 (Cys19Gln20->Ala19Glu20), SFL2647 (Glu29Gln30->Ala29Glu30), SFL2646 (Pro22Gln23->Ala22Glu23), SFL2648 (Ser36->Ala36), and the empty pBAD vector used as negative control (SFL2650). The c-di-GMP concentration was analysed using a Cyclic-di-GMP ELISA kit by Cayman Chemicals. The calculations are based on standard curve for c-di-GMP and assaying each sample in duplicates. Statistically significant variance is denoted by asterisks (*** = *p* value < 0.001, ** = *p* value < 0.01, * = *p* value < 0.05). The standard deviation of data from three autonomous experiments is depicted by the error bars.

The YfiB site-directed mutant strains showed varied c-di-GMP concentrations, presumably due to the amino acid mutations affecting the functioning of YfiB (Figure 8) [29,30]. The observed c-di-GMP concentrations were: 86 pg/mL in SFL2645 (Cys19Gln20->Ala19Glu20); 150 pg/mL in SFL2646 (Pro22Gln23->Ala22Glu23); 108 pg/mL in SFL2647 (Glu29Gln30->Ala29Glu30); and 121 pg/mL in SFL2648 (Ser36->Ala36). The empty pBAD_Myc_HisA vector strain SFL2650 (SFL2641/ΔYfiB strain containing the empty pBAD_Myc_HisA plasmid) used as a negative control had an 85 pg/mL concentration of native c-di-GMP, which was similar to the KO mutant SFL2641/ΔYfiB strain, as seen in Figure 2).

**Figure 9:**
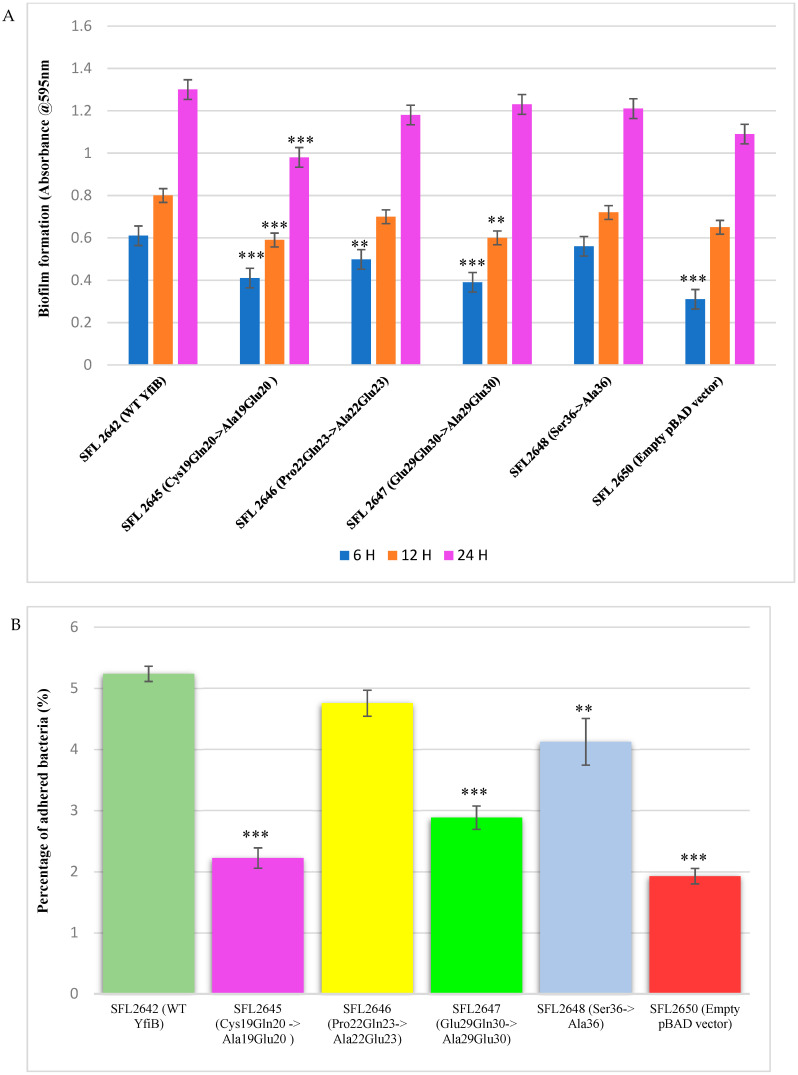
The consequence of YfiB linker mutants and Ser36 mutant cloned in pBAD_*Myc*_HisA vector on biofilm formation and adherence to BHK cells. (**A**) The bar graphs display the biofilm formation of WT YfiB (SFL2642), empty pBAD_Myc_HisA vector (SFL2650), along with YfiB mutants SFL2645 (Cys19Gln20->Ala19Glu20), SFL2646 (Pro22Gln23->Ala22Glu23), SFL2647 (Glu29Gln30->Ala29Glu30), and SFL2648 (Ser36->Ala36) at 6, 12, and 24 h. (**B**) The BHK cells were infected with the WT YfiB protein strain (SFL2642), the empty pBAD_*Myc*_HisA vector strain (SFL2650), along with the YfiB site-directed mutants SFL2645 (Cys19Gln20->Ala19Glu20), SFL2646 (Pro22Gln23->Ala22Glu23), SFL2647 (Glu29Gln30->Ala29Glu30), and SFL2648 (Ser36->Ala36). The percentage of adhered bacteria was calculated by dividing the total CFU of adhered bacteria by the total CFU of the inoculum. The Y-axis represents the percentage of adhered bacteria to BHK cells after 2 h post infection at 37 °C/5% CO_2_. To compute the variance seen in the biofilm production and percentage adhesion between the strains, a Student’s t-test was performed. Statistically significant variance is denoted by asterisks (*** = *p* value < 0.001 and ** = *p* value < 0.01). The standard deviation of data from three autonomous experiments is depicted by the error bars.

Biofilm formation assay was performed with the wild-type YfiB protein expressing strain (SFL2642), along with the YfiB site-directed mutant strains SFL2645 (Cys19Gln20->Ala19Glu20), SFL2646 (Pro22Gln23->Ala22Glu23), SFL2647 (Glu29Gln30->Ala29Glu30), and SFL2648 (Ser36->Ala36). The empty pBAD_*Myc*_HisA vector strain SFL2650 was used as the negative control. The assay was performed at 6, 12, and 24 h, and it was observed that there was a noteworthy difference between the biofilm formation of the wild-type YfiB protein and the functional mutants of YfiB (Figure 7). SFL2645 (Cys19Gln20->Ala19Glu20) mutant showed a decrease in biofilm at all three time points, whereas the SFL2646 (Pro22Gln23->Ala22Glu23) and SFL2647 (Glu29Gln30->Ala29Glu30) mutants only showed a decrease in the biofilm produced at 6 and 12 h. The mutant SFL2648 (Ser36->Ala36) displayed only a slight decrease when compared to the wild-type YfiB strain, but this difference was not statistically significant (Figure 9A).

Bacterial adhesion assay was carried out using the BHK cell line, as described in the methods. BHK monolayers were infected with the *Shigella* strains: WT YfiB protein (SFL2642), the empty pBAD_*Myc*_HisA vector strain (SFL2650), along with the YfiB site-directed mutants SFL2645 (Cys19Gln20->Ala19Glu20), SFL2646 (Pro22Gln23->Ala22Glu23), SFL2647 (Glu29Gln30->Ala29Glu30), and SFL2648 (Ser36->Ala36). It was observed that the YfiB site-directed mutants showed a significantly reduced capacity to adhere to the epithelial cells of the host when compared with the wild-type YfiB protein. Mutants SFL2645 (Cys19Gln20->Ala19Glu20), SFL2647 (Glu29Gln30->Ala29Glu30), and SFL2648 (Ser36->Ala36) showed a significant reduction in the percentage adhesion to BHK cells. Whereas, mutant SFL2646 (Pro22Gln23->Ala22Glu23), showed a slight decrease when compared to the wild type but the difference was not statistically significant (Figure 9B). It can be concluded from these functional assays that the conserved amino acid pairs in the linker sequence (Cys19Gln20, Pro22Gln23, and Glu29Gln30) and the serine at position 36, part of an important amino acid cluster of YfiB (35–55), are assumingly crucial for the activation and proper functioning of the YfiB protein. However, additional structural assessment of YfiB protein in *Shigella* can validate these observations and expand our knowledge of all the critical amino acid residues in YfiB with their respective functions.

## 4. Discussion

In this work, we show that deletion of *yfiB* leads to altered intracellular c-di-GMP levels, which can severely affect the pathogenesis of *Shigella flexneri*. This study suggests that the loss of outer-membrane YfiB protein hinders the function of the inner membrane-bound YfiN (DGC activity), as the periplasmic YfiR is always bound to it, which causes a decreased concentration of c-di-GMP. KO of *yfiB* and the apparent decrease in intracellular c-di-GMP levels subsequently affect other downstream virulence factors of the bacteria, as indicated by a slower biofilm production; decreased adhesion, and invasion of host cells; weakened ability to form plaques; and lower accumulation in guts of *C. elegans*, which leads to a surge in the lifespan of the worms. This decrease in c-di-GMP concentration caused by the *yfiB* KO was also confirmed by quantifying the native c-di-GMP of the KO mutant and wild-type strain along with the complement strain (Figure 2). YfiB acts as a positive regulator of YfiN (a membrane-bound diguanylate cyclase), accountable for the production of c-di-GMP [70]. When YfiB is absent, it cannot sequester YfiR (acting as the negative regulator of YfiN) to the outer membrane and, as a result, YfiN is always bound to YfiR. This causes YfiN to remain in an inactivated state, not being able to produce c-di-GMP and affecting various virulence functions in *Shigella* [28,31,32,71]. In a similar study regarding the importance of c-di-GMP regulation in *Shigella* virulence, conducted by Ojha R. et al. (2021), it is demonstrated that cloning an active diguanylate cyclase (DGC) gene from *Vibrio* in *S. flexneri* elevated the native c-di-GMP levels, which further led to an increase in the biofilm production [20]. Conversely, DGC *Shigella* mutant strains also showed altered virulence phenotypes such as reduced biofilm formation and host cell invasion [20].

Even though biofilm production in bacteria is dependent on a multitude of cellular factors and coordinated pathways, the slow biofilm production seen in the *yfiB* KO mutant can be explained by the functioning of the *yfiBNR* operon model, previously studied in *P. aeruginosa* [26,27]. Disrupting the YfiBNR system by deleting the *yfiB* gene leads to a decreased c-di-GMP level, which is needed by *Shigella* for effective biofilm production in the presence of bile salts [19,21,37]. Higher intracellular levels of c-di-GMP are known to be responsible for an escalation of biofilm production and motility in bacteria [21,22,23,24,25,26,27]. However, in *E. coli*, it was observed that deleting genes encoding diguanylate cyclases such as *yfiN,* causing lower c-di-GMP levels, leads to higher motility and increased early biofilm production [70]. Motility affects biofilm formation as it has a functional role in preliminary surface attachment and attachment to the neighboring bacterial cells [21,22,70]. Bacterial motility also affects the architecture of biofilms, as it is known that strains with reduced motility produce flat biofilms, while strains with prominent motility form vertical biofilm structures [14,21]. This increased early biofilm formation observed in *E. coli* is not similarly observed in *Shigella*, presumably because they are nonmotile bacteria and the decline in c-di-GMP directly decreases the biofilm formation. Earlier experiments in *E. coli* have proven that nonflagellated or bacterial cells with paralyzed flagella show decreased biofilm production in a lower concentration of c-di-GMP [70,72], which confirms the observed slow biofilm production in *Shigella-yfiB* KO mutant.

The effect of *yfiB* KO on *Shigella’*s ability to adhere, invade, and spread in the host cells can also be explained by the decreased intracellular levels of c-di-GMP in the KO mutant. c-di-GMP signalling is known to be interrelated to various bacterial virulence phenotypes such as host cell invasion, secretion of virulence factors, cytotoxicity, intracellular infection, host-cell adherence, cell motility, alteration of immune responses, and resistance to oxidative stress [21,22,23,24,25,26,27]. Invasion and injecting bacterial effectors into the host cells, which is mediated by the type 3 secretion system in *Shigella,* is also affected by the cellular concentration of c-di-GMP [22,73]. High concentrations of c-di-GMP are known to have a progressive effect on the adhesion and invasion of the bacteria, which eventually aids the pathogen in colonizing the host epithelial cells [21,22,23,24,25,26,27]. This relationship between biotic or abiotic surface attachment and c-di-GMP concentration has been previously inspected in the opportunistic human pathogen *P. aeruginosa* [31,32,74] and recently in *S. flexneri* [20]. It is known from these studies that on surface contact, there is an upsurge in levels of c-di-GMP, which induces flagella/pilli biosynthesis and surface adherence and boosts virulence [22]. This explains how by deleting the *yfiB* gene and obstructing the proper functioning of the *yfiBNR* operon, we can severely affect the pathogenicity of *Shigella* by impeding its adhesion, invasion, and intercellular spreading capabilities.

The *C. elegans* accumulation assay to test changes in *Shigella* virulence after deleting *yfiB* gene also validated the finding that YfiB acts as a positive regulator of YfiN producing c-di-GMP. Low accumulation of *yfiB* KO mutant in the *C. elegans* intestinal lumen indicates that it becomes less virulent, eventually increasing the lifespan and survival of worms in comparison to the wild-type strain. This shows how YfiB and the YfiBNR system have a significant impact on the intestinal accumulation of *S. flexneri* in the guts of *C. elegans* worms. It is known that several bacterial species and nonvirulent bacteria, unable to persistently colonize the nematode gut, are rapidly digested and expelled from the intestine [46,47,75]. c-di-GMP catabolizing enzymes have been previously shown, supporting intestinal gut colonization by the bacterium, and improving its intracellular survival [76,77]. As discussed earlier, c-di-GMP plays a significant role in bacterial adherence and invasion processes, which are necessary steps in invading the *C. elegans* gut lumen [21,22,23,24,25,26,27]. Hence, the low bacterial accumulation seen of the *yfiB* KO mutant in *C. elegans* gut is consistent with the other observations of slow biofilm production, reduced adherence and invasion, and decreased plaque numbers, when there is decreased c-di-GMP concentration inside the cells owing to the disruption of *yfiBNR* operon caused by the KO of the *yfiB* gene.

Mutational evaluation of the YfiB protein in this study showed how the conserved amino acid pairs of the linker sequence and the conserved serine at position 36 are essential for its proper functioning. The four YfiB mutants—SFL2645 (Cys19Gln20->Ala19Glu20), SFL2647 (Glu29Gln30->Ala29Glu30), SFL2646 (Pro22Gln23->Ala22Glu23), and SFL2648 (Ser36->Ala36), along with the WT YfiB protein (SFL2642) and the pBAD_*Myc*_HisA empty vector (SFL2650) used as negative control, were subjected to a biofilm formation assay and bacterial adhesion assay to assess phenotypic differences caused by the mutations. Firstly, protein expression was confirmed to check if the amino acid mutation caused expression defects. Western blot was conducted using the anti-HisA antibody and it was seen that all the YfiB mutant proteins were expressed appropriately in the *Shigella* strains. The linker sequence acts as a lipid anchor and helps in peptidoglycan binding, which is critical for the full activity of the YfiB [29,30]. Anchoring of YfiB in the cell wall and outer membrane aids in proper signalling and accurate YfiR sequestration, which eventually activates the YfiN, leading to c-di-GMP production [29,30,31,32]. Mutations of the conserved amino acid pairs of the linker sequence result in a decreased c-di-GMP level, possibly due to improper functioning of the YfiB and the YfiBNR system as seen in Figure 8 [29,30]. This is then shown to have a significant effect on all downstream virulence-related progressions in bacteria such as the formation of biofilm, attachment, and invasion of the host cells, to name a few, which are regulated by c-di-GMP [26,27,29,30,31,32]. The serine at position 36 was also found to be conserved in most YfiB homologs and had the highest frequency of being present at this position when compared with other similar amino acids (Supplementary Figure S2B). This was mutated to alanine to test its function (SFL2648 Ser36->Ala36) and it was observed that it does not exhibit any difference in biofilm production, but it demonstrates a significant decrease in the percentage adhesion to BHK cells when compared to the wild-type YfiB. Serine at position 36 is part of an important amino acid cluster of YfiB (35–55), which is known to be involved in the activation of YfiB protein, proper YfiR sequestration, and considerably effects c-di-GMP production levels, as seen in this study (Figure 8) [29,30]. This mutational analysis demonstrated the importance of the conserved amino acid residues, especially the ones present in the linker sequence, as they are significantly involved in YfiB functioning. The effect on c-di-GMP levels disrupting proper YfiB signalling by amino acid mutations proves that YfiN is under positive and negative control of YfiB and YfiR, respectively, and together, this YfiBNR system regulates the intracellular quantities of c-di-GMP, which aids in various virulence mechanisms of the bacteria.

Each year, *Shigella* is known to cause millions of infections globally and it has become a major public health concern. There is a gradual decrease of treatment alternatives as a result of the rise in antimicrobial resistance and the lack of *Shigella* vaccine which could prevent infections [1,2,3,4,5,6,7,8,9]. Hence, it has become extremely important to understand every aspect of the *Shigella* infection model to develop newer drug targets and possible vaccine candidates. This present study establishes that the c-di-GMP regulation by the YfiBNR system is involved in *S. flexneri’s* in vivo persistence, biofilm formation, and its infection cycle. c-di-GMP is a well-known player in bacterial virulence, promoting prolonged survival of the pathogen and, as homologs of YfiBNR exist in other Gram-negatives, this regulatory system can be used as an effective drug target in treating bacterial diseases [78,79,80]. Developing antibacterial drugs that can affect the functioning of YfiB or YfiN directly and/or other diguanylate cyclase present in the bacteria could result in effectively controlling the spread and decreasing the span of bacterial infections [78,79,80].

This advance could also reduce our dependency on antimicrobials to treat infections and the risk of generating any multidrug-resistant strains. This study provides a starting point for mechanistic and functional research of the YfiBNR signalling system and c-di-GMP mediated virulence in *Shigella*. Furthermore, it inspects the prospect of targeting this signaling network, for discovering newer therapeutics which could be used against various bacterial infections such as shigellosis.

## Data Availability

Additional data are provided in the Supplementary Material.

## Supplementary Materials

The following supporting information can be downloaded at: https://www.mdpi.com/article/10.3390/microorganisms10030653/s1. Figure S1. Results of conserved domain analysis, Clustal Omega alignment of YfiB from *Pseudomonas* and *Shigella*, and predicted protein structure by I- TASSER. (a) Conserved domains found in the YfiB protein, analysed using NCBI-protein BLAST and conserved domain database (CDD). (b) Clustal Omega sequence alignment of YfiB protein from *Pseudomonas* and *Shigella*. Highlighted sequences indicate: Blue—conserved linker sequence; Purple—35–55 amino acid cluster important for activating YfiB; Yellow—PAL-Domain-associated amino acids. Symbols denoting ***** conserved amino acid residue,: strongly similar amino acid residue and. weakly similar amino acid residues (c) The 3D protein structure predicted for YfiB, computed by the I-TASSER tool, using *Pseudomonas* YfiB solved crystal structure as a template; the 3D structure consists of Core OmpA-like domain, four-stranded antiparallel β-sheet with topology β1-β4-β2-β3 and helices α1–3. Figure S2. The in silico investigation of the YfiB protein. (a) Clustal Omega multiple-sequence alignment of YfiB protein homologs found in various Gram-negative bacteria, showing conserved and non-conserved amino acid residues. (b) Weblogo representations of the YfiB protein amino acid conservation. The height of amino acid code at each position reflects the comparative incidence of the amino acid at that position, and the total height of the pile denotes the degree of conservation at each individual locus (measured in bits). The consensus sequence was derived from multiple-sequence alignment of YfiB protein sequences from various Gram-negative bacteria. (c) Phylogenetic tree illustrating the evolutionary distance between the YfiB protein homologs from various Gram-negative bacteria. Clustal Omega alignment of the homologs was used to generate this phylogenetic tree and was created using the MEGA software, calculated by the Maximum Composite Likelihood method. Figure S3. Confirming protein expression of WT and mutant YfiB proteins. (a) The sequence of the wild-type N-terminal linker and the three site-directed linker mutants. The amino acid pairs mutated are highlighted in red. (b) The sequence of the wild-type YfiB-activating domain with serine at position 36 and the mutant sequence with alanine at position 36, highlighted in red. (c) SDS gel stained with Coomassie blue dye for validating equal total protein loading for each sample. (d) Western transfer and blotting of the loaded SDS gel using the anti-HisA antibody to check for correct protein expression. YfiB is known to be a 17.2 kDa protein, WT YfiB protein sample and site-directed mutants of YfiB all show a band at the same location, above the 15 kDa band of the protein ladder. * Lane 1—Pre-stained protein ladder (10–250 kDa); Lane 2—SFL2650 (empty pBAD_*Myc*_HisA vector); Lane 3—WT YfiB (SFL2642/YfiBComp); Lane 4—SFL2645 (Cys19Gln20->Ala19Glu20); Lane 5—SFL2646 (Pro22Gln23->Ala22Glu23); Lane 6—SFL2647 (Glu29Gln30->Ala29Glu30); and Lane 7—SFL2648 (Ser36->Ala36). Table S1. Bacterial strains and plasmids used in this study. Table S2. Primers used in this study. Table S3. UniProt accession number of the YfiB protein sequences used in this study for in silico analysis.
