# Supplementary material for: YfiB: An Outer Membrane Protein Involved in the Virulence of Shigella flexneri
Source: Microorganisms. 2022 Mar 18;10(3):653. doi: 10.3390/microorganisms10030653 (PMC8948675; doi:10.3390/microorganisms10030653)

Supplementary Figure S1. Results for conserved domain analysis, Clustal-Omega alignment of YfiB from *Pseudomonas* and *Shigella*, and predicted protein structure by I-TASSER. (a) Conserved domains found in the YfiB protein, analysed using NCBI-protein BLAST and conserved domain database (CDD). **(b)** Clustal-Omega sequence alignment of YfiB protein from *Pseudomonas and Shigella*. Highlighted sequences indicate: Blue- conserved linker sequence; Purple- 35-55 amino acid cluster important for activating YfiB; Yellow - PAL Domain associated amino acids. Symbols denoting- : strongly similar amino acid residue; . weakly similar amino acid residue; * conserved amino acid residue. **(c)** The 3D protein structure predicted for YfiB, computed by the I-TASSER tool, using *Pseudomonas* YfiB solved crystal structure as a template; the 3D structure consists of Core OmpA-like domain, four-stranded anti- parallel β-sheet with topology β1-β4-β2-β3 and helices α1-3.

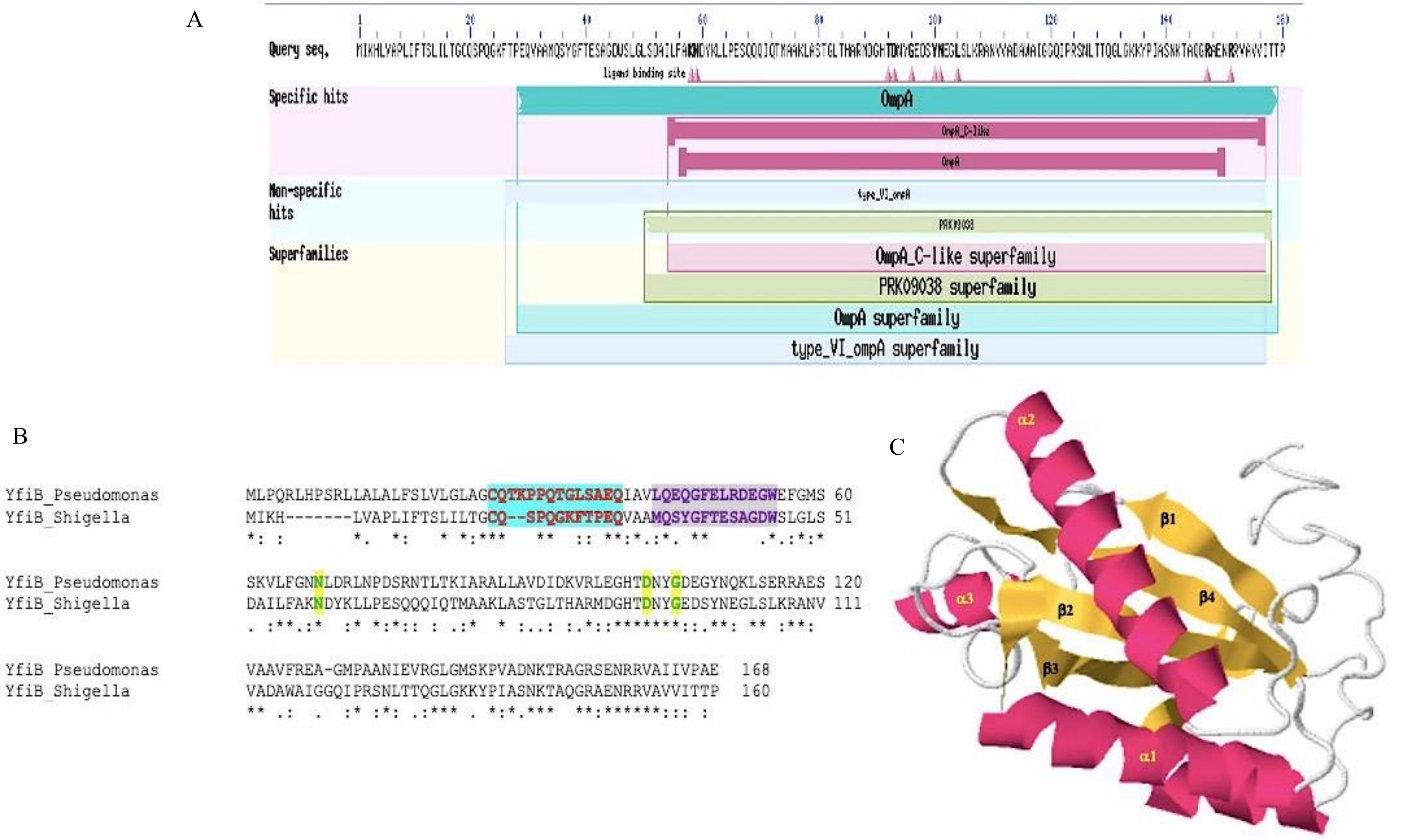

Supplement: Supplementary file 1 [file Supplementary figure S1.pdf › Supplementary figure S1.pdf]
