# Supplementary material for: YfiB: An Outer Membrane Protein Involved in the Virulence of Shigella flexneri
Source: Microorganisms. 2022 Mar 18;10(3):653. doi: 10.3390/microorganisms10030653 (PMC8948675; doi:10.3390/microorganisms10030653)

Supplementary Figure S2. The *in-silico* investigation of the YfiB protein. (A) Clustal-Omega multiple sequence alignment of YfiB protein homologs found in various Gram-negative bacteria, showing conserved and non-conserved amino acid residues.

A

```
YfiB_Citrobacter                    --------------------------------------------MKSYGF
YfiB_Enterobacter                   ---------MLKRYFAPLLLASLVISGCQT-SPEGKFTPEQIAAMKSYGF
YfiB_E.coli                         ---------MIKHLVAPLIFTSLILTGCQ--SPQGKFTPEQVAAMQSYGF
YfiB_S.dysenteriae                  ---------MIKHLVAPLIFTSLILTGCQ--SPQGKFTPEQVAAMQSYGF
YfiB_S.flexneri_X                   ---------MIKHLVAPLIFTSLILTGCQ--SPQGKFTPEQVAAMQSYGF
YfiB_S.sonnei                       ---------MIKHLVAPLIFTSLILTGCQ--SPQGKFTPEQVAAMQSYGF
YfiB_SFL1613/Y394                   ---------MIKHLVAPLIFTSLILTGCQ--SPQGKFTPEQVAAMQSYGF
YfiB_S.boydii                       --------------------------------------------MQSYGF
YfiB_Klebsiella_pneumoniae          ---------MIRKYFVPALMAAALLTGCQ--APQGKFTPEQVAAMKSYGF
YfiB_Xenorhabdus                    ------MSKKRSFMFICAFIGTLFLSACQ---NKGGLTAEQITTLKQQGF
YfiB_Pseudomonas_chlororaphis       ----------MQLFTAGLLLALLSLTGCQSVPP-KGLTPQQVAVLKQEGF
YfiB_Pseudomonas_aeruginosa         --MLPQRLHPSRLLALALFSLVLGLAGCQTKPPQTGLSAEQIAVLQEQGF
YfiB_Yersinia_enterocolitica        MLGLNNNRQKNPLWISFFALCLLVLVGCQAKPH-QGLTPEQIAALQEQGF
YfiB_Serratia                       --MIQQTLKNRFSLLAMMFIALLALAGCQSKPQ--GLTPEQIALLQSQGF
YfiB_Aeromonas                      ------------------MGMFALLLAGCQ-SPPAGRLNETQIALLKARGF
YfiB_Acinetobacter_baumannii        ------------MKLSFIALLCIALAGCLS---FGPLKYRQVKMLKKEGF
                                                                                ::  **

YfiB_Citrobacter                    TEASGDWSLGLSDNILFDKNDYKLRPESEKQIKEMASKLAATGLNHARLD
YfiB_Enterobacter                   NELNGDWSLGLSDKILFDKNDARLRPESETQIQTMASRLAATGLNHARMD
YfiB_E.coli                         TESAGDWSLGLSDAILFAKNDYKLLPESQQQIQTMAAKLASTGLTHARMD
YfiB_S.dysenteriae                  TESAGDWSLGLSDAILFAKNDYKLLPESQQQIQTMAAKLASTGLTHARMD
YfiB_S.flexneri_X                   TESAGDWSLGLSDAILFAKNDYKLLPESQQQIQTMAAKLASTGLTHARMD
YfiB_S.sonnei                       TESAGDWSLGLSDAILFAKNDYKLLPESQQQIQTMAAKLASTGLTHARMD
YfiB_SFL1613/Y394                   TESAGDWSLGLSDAILFAKNDYKLLPESQQQIQTMAAKLASTGLTHARMD
YfiB_S.boydii                       TESAGDWSLGLSDAILFAKNDYKLLPESQQQIQTMAAKLTSTGLTHARMD
YfiB_Klebsiella_pneumoniae          TESNGDWSLGLSDSILFDKNDYRLRPDSRQQITTMASRLAATGITHSRLE
YfiB_Xenorhabdus                    QQTDEGWLFGMSEKVLFGNNQSHLRPEGEAKLKELASVLSKVGIHHARLD
YfiB_Pseudomonas_chlororaphis       ELTDEGWAFGLSGKVLFGSDIETLNAASTEIVERIGKALVSVHIDKVRVD
YfiB_Pseudomonas_aeruginosa         ELRDEGWEFGMSSKVLFGNNLDRLNPDSRNTLTKIARALLAVDIDKVRLE
YfiB_Yersinia_enterocolitica        KLTDNGWVFGLANKVLFDSDVRKLNASGVQTVQNIGRALHNVGINHMRVD
YfiB_Serratia                       KLTDNGWEFGLSDKVLFGNNVGKLNPESTDTVQKMGRALMSVGITQFRLD
YfiB_Aeromonas                      TQVEEGWSLGLSDRVLFANESRTLNPDSRAVVQKIAHSLLSVGIDWARLD
YfiB_Acinetobacter_baumannii        VLTNEGWTLGLPERLLFDFNDATLKQSHEAELTRLANQLNKYDLNKLKIV
                                         .* :*:.  :**  :   *       :  :.  *    :   ::

YfiB_Citrobacter                    GHTDNYGEDSYNEALSLKRANVVADAWAQGANIPRTNLTTQGLGKKYPIA
YfiB_Enterobacter                   GHTDNYGEESYNEALSLKRANVVADAWAKGANIPRSNLTTRGLGKKYPVS
YfiB_E.coli                         GHTDNYGEDSYNEGLSLKRANVVADAWAMGGQIPRSNLTTQGLGKKYPIA
YfiB_S.dysenteriae                  GHTDNYGEDSYNEGLSLKRANVVADAWAMGGQIPRSNLTTQGLGKNIP--
YfiB_S.flexneri_X                   GHTDNYGEDSYNEGLSLKRANVVADAWAIGGQIPRSNLTTQGLGKKYPIA
YfiB_S.sonnei                       GHTDNYGEDSYNEGLSLKRANVVADAWAIGGQIPRSNLTTQGLGKKYPIA
YfiB_SFL1613/Y394                   GHTDNYGEDSYNEGLSLKRANVVADAWAIGGQIPRSNLTTQGLGKKYPIA
YfiB_S.boydii                       GHTDNYGEDSYNEGLSLKRANVVADAWAMGGQIPRSNLTTQGLGKKYPIA
YfiB_Klebsiella_pneumoniae          GHTDNYGEDSYNEALSLKRANSVADAWAEGAHVPRSNLVTRGLGKKIPDR
YfiB_Xenorhabdus                    GHTDNYGEVSYNNQLSLKRANTVADALTDGG-MQRANLTTRGLGPSQPIA
YfiB_Pseudomonas_chlororaphis       GHTDASGREAYNQQLSLRRAKSVSKVLVATG-MREENIQLRGLGSSEPVA
YfiB_Pseudomonas_aeruginosa         GHTDNYGDEGYNQKLSERRAESVAAVFREAG-MPAANIEVRGLGMSKPVA
YfiB_Yersinia_enterocolitica        GHTDAIGEDGYNQQLSFQRASAVADTLAAIG-IPRTNIEVRGRGKLEPVA
YfiB_Serratia                       GHTDNYGEDSYNDQLSLRRANAVADLLASVG-IPRANIETRGMGKRDPVA
YfiB_Aeromonas                      GHTDSNGDESYNQQLSRQRAQSVADSLIDAG-MPVANLEVRGLGERYPVA
YfiB_Acinetobacter_baumannii        GHTDDVGNPEYNQKLSEERAQSVANLFLTHG-FKKENIYVIGRGSTQPYV
                                    ****  *   **: ** .**. *:      . .   *:   * *   *

YfiB_Citrobacter                    SN------------QTSKGRAENRRVAVVITTP-----------------
YfiB_Enterobacter                   SN------------HTAQGRAENRRVAVVISTP-----------------
YfiB_E.coli                         SN------------KTAQGRAENRRVAVVITTP-----------------
YfiB_S.dysenteriae                  --------------------------------------------------
YfiB_S.flexneri_X                   SN------------KTAQGRAENRRVAVVIATP-----------------
YfiB_S.sonnei                       SN------------KTAQGRAENRRVAVVITTP-----------------
YfiB_SFL1613/Y394                   SN------------KTAQGRAENRRVAVVITTP-----------------
YfiB_S.boydii                       SN------------KTAQGRAENRRVAVVITTP-----------------
YfiB_Klebsiella_pneumoniae          QQRHGGRARRKPPGNGGHQHAVSLRLVAGLHHPHCRCRAPAQVVNGNEQQ
YfiB_Xenorhabdus                    DN------------RSSKGRAENRRVAIVITAP-----------------
YfiB_Pseudomonas_chlororaphis       SN------------STVAGRTENRRVSIVVIAD-----------------
YfiB_Pseudomonas_aeruginosa         DN------------KTRAGRSENRRVAIIVPAE-----------------
YfiB_Yersinia_enterocolitica        DN------------RTAKGRAENRRVAIIVTAP-----------------
YfiB_Serratia                       DN------------RTSSGRVENRRVAIVVTP------------------
YfiB_Aeromonas                      SN------------KTREGRSQNRRVAIVITD------------------
YfiB_Acinetobacter_baumannii        PN------------TTNENRAINRRVAIVIIP------------------

YfiB_Citrobacter                    -----
YfiB_Enterobacter                   -----
YfiB_E.coli                         -----
YfiB_S.dysenteriae                  -----
YfiB_S.flexneri_X                   -----
YfiB_S.sonnei                       -----
YfiB_SFL1613/Y394                   -----
YfiB_S.boydii                       -----
YfiB_Klebsiella_pneumoniae          QAGSH
YfiB_Xenorhabdus                    -----
YfiB_Pseudomonas_chlororaphis       -----
YfiB_Pseudomonas_aeruginosa         -----
YfiB_Yersinia_enterocolitica        -----
YfiB_Serratia                       -----
YfiB_Aeromonas                      -----
YfiB_Acinetobacter_baumannii        -----
```

Supplementary Figure S2. The *in-silico* investigation of the YfiB protein. (b) Weblogo representations of the YfiB protein amino acid conservation. The height of amino acid code at each position reflects the comparative incidence of the amino acid at that position and the total height of the pile denotes the degree of conservation at each individual locus (measured in bits). The consensus sequence was derived from multiple sequence alignment of YfiB protein sequences from various Gram-negative bacteria**.**

B

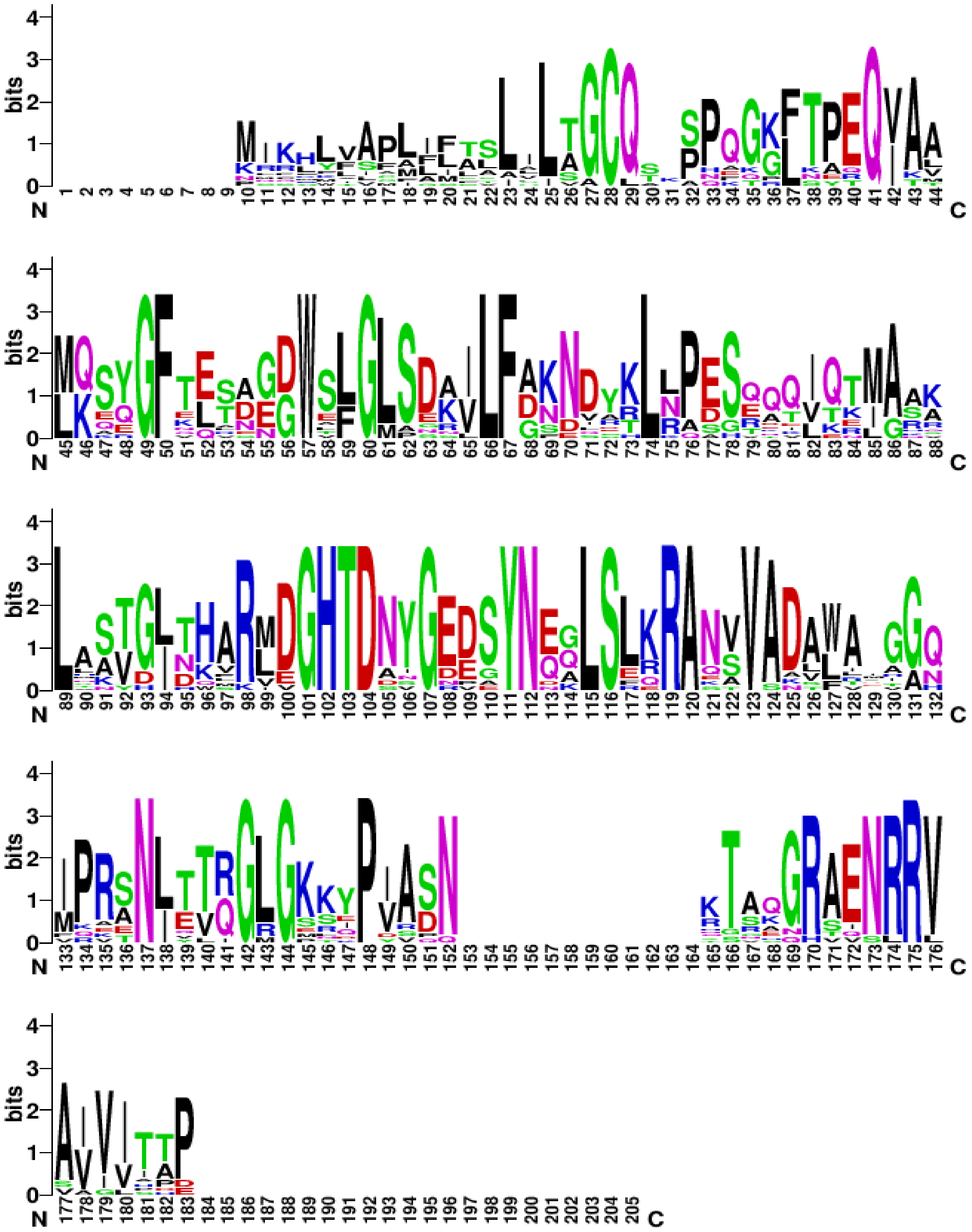

Supplementary Figure S2. The *in-silico* investigation of the YfiB protein. (c) Phylogenetic tree illustrating the evolutionary distance between the YfiB protein homologs from various gram-negative bacteria. Clustal-Omega alignment of the homologs was used to generate this phylogenetic tree and was created using the MEGA software, calculated by the Maximum Composite Likelihood method.

C

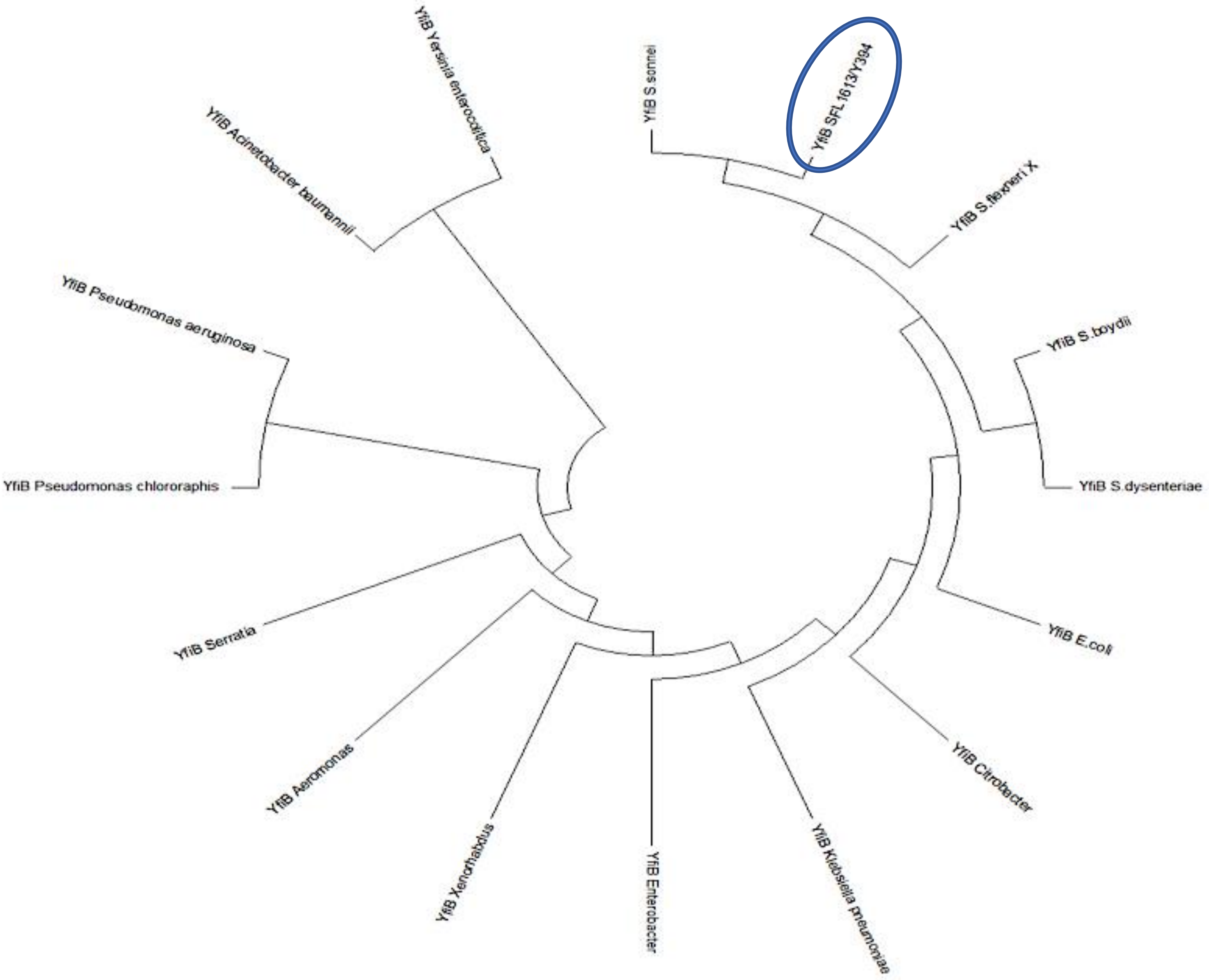

Supplement: Supplementary file 1 [file microorganisms-10-00653-s001.zip › Supplementary Figure S2 (A-C).pdf]
