# Supplementary material for: YfiB: An Outer Membrane Protein Involved in the Virulence of Shigella flexneri
Source: Microorganisms. 2022 Mar 18;10(3):653. doi: 10.3390/microorganisms10030653 (PMC8948675; doi:10.3390/microorganisms10030653)

Supplementary Figure S3. Confirming protein expression of WT and mutant YfiB proteins. (a) The sequence of the wildtype N-terminal linker and the three site-directed linker mutants. The amino acid pairs mutated, are highlighted in red. **(b)** The sequence of the wildtype YfiB activating domain with serine at position 36 and the mutant sequence with alanine at position 36, highlighted in red. **(c)** SDS gel stained with Coomassie blue dye for validating equal total protein loading for each sample. **(d)** Western transfer and blotting of the loaded SDS gel using the anti-HisA antibody to check for correct protein expression. YfiB is known to be a 17.2 kDa protein, WT YfiB protein sample and site directed mutants of YfiB, all show a band at the same location, above the 15 kDa band of the protein ladder. *Lane 1- Pre-stained protein ladder (10-250 kDa); Lane 2- SFL2650 (empty pBAD_*Myc*_HisA vector); Lane 3- WT YfiB (SFL2642/YfiBComp); Lane 4- SFL2645 (Cys19Gln20->Ala19Glu20); Lane 5- SFL2646 (Pro22Gln23->Ala22Glu23); Lane 6- SFL2647 (Glu29Gln30->Ala29Glu30); & Lane 7- SFL2648 (Ser36->Ala36).

A

WT YfiB Linker (SFL2642)	Mutant Linker (SFL 2645)	Mutant Linker (SFL 2646)	Mutant Linker (SFL 2647)
**CQSPQGKFTPEQ**	**AESPQGKFTPEQ**	**CQSAEGKFTPEQ**	**CQSPQGKFTPAE**

B

WT conserved YfiB activating domain (SFL2642)	Mutant YfiB activating domain (SFL2648)
**MQSYGFTESAGDW**	**MQAYGFTESAGDW**

C

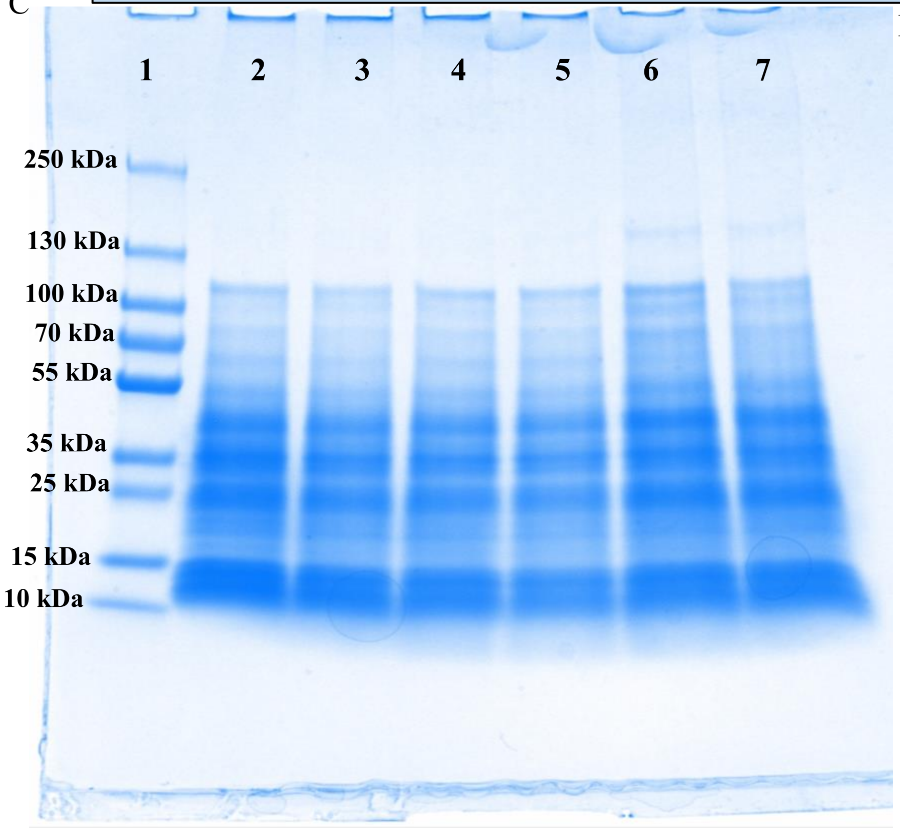

D

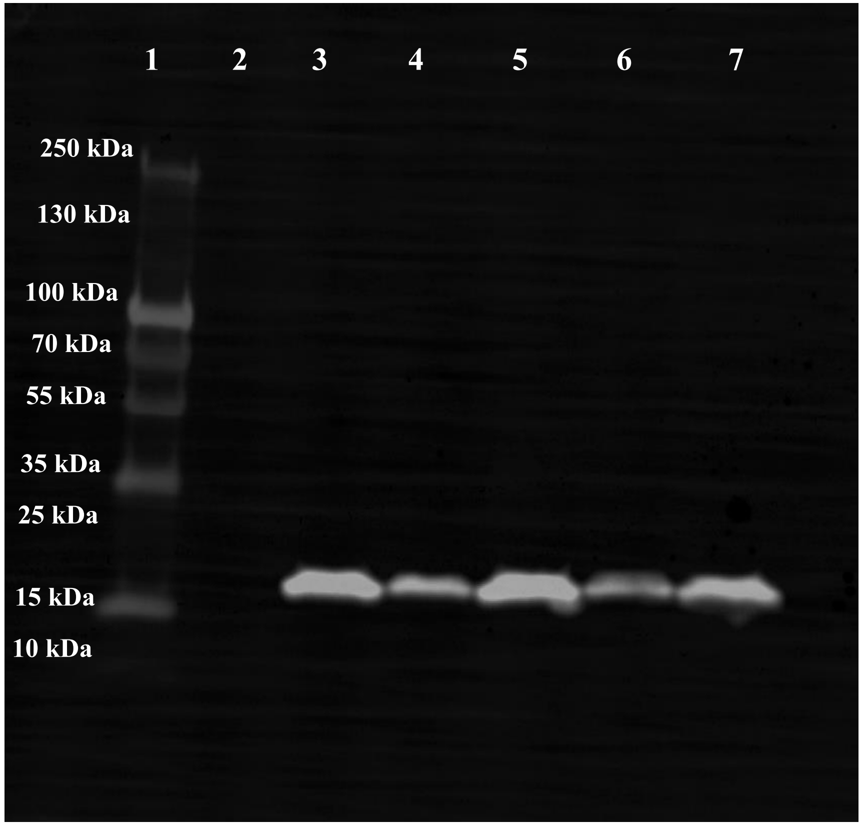

Supplement: Supplementary file 1 [file microorganisms-10-00653-s001.zip › Supplementary figure S3 (A-D).pdf]
