# Supplementary material for: YfiB: An Outer Membrane Protein Involved in the Virulence of Shigella flexneri
Source: Microorganisms. 2022 Mar 18;10(3):653. doi: 10.3390/microorganisms10030653 (PMC8948675; doi:10.3390/microorganisms10030653)
Supplement: Supplementary file 1 [file microorganisms-10-00653-s001.zip › Supplementary Tables (S1-S3).pdf]

Supplementary materials

YfiB: An Outer Membrane Protein Involved in the Virulence of *Shigella flexneri*

Tanuka Sen (1), Naresh K Verma (1)*

Authors affiliation

Division of Biomedical Science and Biochemistry, Research School of Biology, The Australian National University, ACT, Canberra, Australia.

Corresponding author: Naresh K. Verma.

This file includes Tables S1, S2, and S3.

Table S1- Bacterial strains and plasmids used in this study.

Name	Description	Antibiotic Resistance
Shigella flexneri		
SFL1613/Y394	*S.flexneri 1c strain*[33]	-
SFL2608 (1c WT)	SFL1613/Y394 with pKD46 helper plasmid. Wildtype used for this study.	Amp
SFL2641/ΔYfiB	SFL2606 (1c WT) with *yfiB* gene deleted	CM
SFL2642/YfiBComp	SFL2641 with *yfiB* gene complemented, cloned into pBAD_Myc_HisA vector	Amp, Ery, CM
SFL2645	SFL 2641 with site directed mutant- Cys19Gln20-> Ala19Glu20 , cloned into pBAD_Myc_HisA vector.	Amp, Ery, CM
SFL2646	SFL 2641 with site directed mutant- Pro22Gln23-> Ala22Glu23, cloned into pBAD_Myc_HisA vector.	Amp, Ery, CM
SFL2647	SFL 2641 with site directed mutant- Glu29Gln30 -> Ala29Glu30, cloned into pBAD_Myc_HisA vector.	Amp, Ery, CM
SFL2648	SFL 2641 with site directed mutant- Ser36-> Ala36, cloned into pBAD_Myc_HisA vector.	Amp, Ery, CM
SFL2650	SFL 2641 with empty pBAD_Myc_HIsA vector	Amp, Ery, CM
E. coli		
B2298	OP50 *E. coli* strain	-
**Plasmids**		
pKD46	Helper plasmid expressing the lambda red genes (*gam, beta*, and *exo*)	Amp
pKD3	Used for template generation for homologous recombination.	Cm
pBAD_*Myc*_HisA	Vector used for cloning *yfiB* gene, used as control empty vector.	Amp, Ery
pBAD_*Myc*_HisA_YfiB	pBAD_*Myc*_HisA with *yfiB* gene cloned at the *Nco*I and *Hind*III.	Amp, Ery
pBAD_*Myc*_HisA_M1	pBAD_*Myc*_HisA with mutant YfiB (Cys19Gln20 -> Ala19Gln20)	Amp, Ery
pBAD_*Myc*_HisA_M2	pBAD_*Myc*_HisA with mutant YfiB (Pro22Gln23 -> Ala22Glu23)	Amp, Ery
pBAD_*Myc*_HisA_M3	pBAD_*Myc*_HisA with mutant YfiB (Glu29Gln30 -> Ala29Glu30)	Amp, Ery
pBAD_*Myc*_HisA_M4	pBAD_*Myc*_HisA with mutant YfiB (Ser36-> Ala36)	Amp, Ery

Table S2- Primers used in this study.

Primer Name	Role	Sequence
*yfiB*_For	Primer for amplifying the CM gene of pKD3, containing the 80bp overhangs, homologous to the upstream of the *yfiB* gene	AAGAGCTTGCCGATCACAATATGTATCAGGCCAAACACCAGCGTG<br>CCGAAAAGCTGGTGAGATAACAAGGATATATCGATTACACGTCTT<br>GAGCGATTGT
*yfiB*_Rev	Primer for amplifying the CM gene of pKD3, containing the 80bp overhangs, homologous to the downstream of the *yfiB* gene	GGCTGCTCGTATCAAAGAGCGTCTTAACTAAGATTTCGCTTAAGCGA<br>CATCCTGTTAAGAAGGGCTGGCCAATTGGCTGGGGTCCATATGAA<br>TATCCTCC
*yfiB*_Test_For	Confirming deletion of *yfiB* gene	AGCCGATTCGTCACAATGGT
*yfiB*_Test_Rev	Confirming deletion of *yfiB* gene	CTCCGGTAGTTGACAGCATT
CM_Test	Confirming deletion of *yfiB* gene and presence of chloramphenicol gene	CAACGGTGGTATATCCAGTG
Apy_For	Checking presence of *Shigella*'s Virulence plasmid	CATAATCAAGAGACAAAACGATA
Apy_Rev	Checking presence of *Shigella*'s Virulence plasmid	CCAGCCTTTCCAGTAATCCC

Table S2- Primers used in this study.

VirG_For	Checking presence of *Shigella*'s Virulence plasmid	CGGGTACTCAAGAACTTCAAT
VirG_Rev	Checking presence of *Shigella*'s Virulence plasmid	TTCCGCCAAAATGAGAGTTCC
pBAD_Univ_For	Primer to check for correct cloning in the pBAD_Myc_HisA vector	ATGCCATAGCATTTTTATCC
pBAD_Uni_Rev	Primer to check for correct cloning in the pBAD_Myc_HisA vector	GATTTAATCTGTATCAGG

Table S3- UniProt accession number of the YfiB protein sequences used in this study for in-silico analysis.

**UniProt ID[b]**	**Organism**
C3SYR2	*Escherichia coli*
A0A377W1F8	*Klebsiella pneumoniae*
A0A2X2HQ42	*Shigella boydii*
A0A2X2ISV4	*Shigella dysenteriae*
D2AHI5	*Shigella flexneri* serotype X (strain 2002017)
Q3YYN3	*Shigella sonnei* (strain Ss046)
A0A2I8JE62	*Shigella flexneri 1c* strain (Y394)
Q9I4L6	*Pseudomonas aeruginosa (strain ATCC 15692)*
A0A3G7JRF7	*Pseudomonas chlororaphis subsp. aurantiaca*
A0A7G2IWZ5	*Citrobacter freundii*
A0A7U4NUK4	*Acinetobacter baumannii*
A0A0S2SF47	*Aeromonas schubertii*
G8LNV7	*Enterobacter ludwigii*
N1NGC1	*Xenorhabdus nematophila F1*
A0A1W5DTJ7	*Serratia proteamaculans*
A0A485DSF5	*Yersinia enterocolitica*
